# Gd^3+^-Driven Microstructure Regulation of Tm^3+^, Yb^3+^: Gd_x_Bi_2−x_WO_6_ for High-Sensitivity Temperature Sensing and Magnetism

**DOI:** 10.3390/molecules31193557

**Published:** 2026-10-06

**Authors:** Xin Feng, Linxiang Wang, Jiachen Shi, Mengliang Jiang, Munire Maimaiti, Yan Zhang, Yue Bai

**Affiliations:** 1 School of Physics and Electronic Engineering, Xinjiang Normal University, Urumqi 830054, China; 2 Xinjiang Key Laboratory for Luminescence Minerals and Optical Functional Materials, Xinjiang Normal University, Urumqi 830054, China

**Keywords:** Gd^3+^ ion doping, microstructure, upconversion luminescence, temperature sensing, magnetic ability

## Abstract

Tm^3+^, Yb^3+^: Gd_x_Bi_2−x_WO_6_ series upconversion luminescent materials were synthesized via a solid-state method. Calculations and XRD analysis reveal that Gd^3+^ in the lattice exhibits both substitutional and interstitial doping. Compared with the NaYF_4_ matrix, the Bi_2_WO_6_ material doped with Gd^3+^ has a lower Debye temperature (392.45 K), and the maximum phonon energy is low; thus, the probability of nonradiative transitions decreases. SEM images and EDS results reveal that the powder particle size ranges from 0.5 to 3 μm and that the elements are evenly distributed on the surface. The diffuse reflectance absorption spectra indicate that moderate Tm^3+^, Yb^3+^ and Gd^3+^ doping shifts the absorption band of the material towards longer wavelengths and decreases the band gaps. Under 980 nm laser excitation and a Gd^3+^ doping concentration of x = 0.2, the emission intensities of Tm^3+^ at 476 nm, 683 nm, 703 nm, and 804 nm increase by 1.8, 1.24, 1.35, and 81.9 times, respectively. Calculations reveal that these emissions in the range of 450 to 850 nm originate from three-photon or two-photon absorption. Gd^3+^ codoping can prolong the lifetime of the Tm^3+^ energy levels. When this sample was excited at 980 nm (518 mW), the temperature was characterized by *FIR*_703nm/476nm_ from 298 K to 573 K, and the obtained maximum relative sensitivity was 3.4907 K^−1^ (298 K). The minimum temperature resolution was 0.0083 K (298 K). Compared with that of the undoped Gd^3+^ sample, the temperature sensitivity of the Gd^3+^-codoped sample increased by 92.35 times. Under an external magnetic field of 20 kOe and a temperature of 300 K, the magnetization of the sample doped with Gd^3+^ increased fourfold to 0.08174 emu/g. This material has potential applications in temperature sensing and magnetic resonance imaging.

## 1. Introduction

Rare-earth upconversion nanomaterials with adjustable particle sizes, good photostability, and low background fluorescence interference have broad applications in noncontact environmental temperature measurements [1,2]. Several reports have investigated the upconversion luminescence and temperature measurement properties of different matrix materials codoped with Tm^3+^ and Yb^3+^. For example, Zhou [3] prepared Y_2_O_3_: Yb^3+^, Tm^3+^ via fusion and used the three Stark-split bands of Tm^3+^ to achieve temperature sensing at low and high temperatures with the same material. An [4] prepared a series of Tm^3+^, Yb^3+^: LuAG materials using the floating zone method with a CO_2_ laser and investigated the optical temperature sensing properties in the range of 298–1023 K. The maximum absolute and relative sensitivities for optical temperature sensing were obtained at 1023 K and 555 K, respectively. Additionally, Krishna [5] used a hydrothermal method to dope Tm^3+^ and Yb^3+^ into Ca_3_(PO_4_)_2_. The Judd–Ofelt intensity parameters were calculated and found to follow the order Ω_2_ > Ω_4_ > Ω_6_, and the maximum absolute sensor sensitivity was 0.00363 K^−1^ at 623 K when ^1^G_4_ and ^3^F_2,3_ levels were considered nonthermally coupled.

As reported in the literature [6], Gd^3+^ doping can regulate the luminescent properties of certain luminescent materials by adjusting their energy band structure and changing their lattice symmetry. Zhang [7] prepared a series of AWO_4_ (A = Sr, Ca) materials doped with Gd^3+^, Yb^3+^, and Er^3+^ via a high-temperature solid phase method, and Gd^3+^ doping effectively improved the luminescence and thermometry sensitivity. Furthermore, Xiao [8] synthesized CaF_2_: Gd^3+^, Yb^3+^ and Ho^3+^ using a hydrothermal method and reported that the introduction of Gd^3+^ reduced the clustering of Ho^3+^ and Yb^3+^ and increased their upconversion luminescence efficiency. Yunfeng Bai [9] investigated the effect of Gd^3+^ doping on the luminescence of Lu_3_Al_5_O_12_: 1% Ce^3+^ powders and reported that as the concentration of Gd^3+^ increased, the 5d orbital splitting of Ce^3+^ increased, which led to broadening of the Ce^3+^ emission peak and a redshift in the Ce^3+^ emission. Moreover, Ilya E. Kolesniko [10] prepared NaYF_4_: Eu^3+^ and Gd^3+^ samples via a hydrothermal method using citric acid as a stabilizer and reported that when Gd^3+^ with a relatively large radius replaced Y^3+^, the positive charge density on the crystal surface decreased, and the luminescence increased. S. G. Revankard [11] analysed the upconversion luminescence of materials codoped with Gd^3+^ and a series of Ln^3+^ (Eu^3+^, Tb^3+^, and Dy^3+^). As the energy gap between the P_j_ state and the ground state of Gd^3+^ is too large for the energy to dissipate into the surrounding lattice, energy is transferred to Ln^3+^, increasing the luminescence intensity of Ln^3+^. In summary, Gd^3+^ doping may alter the lattice symmetry and substrate band gap, thus affecting the luminescence and fluorescence temperature measurement properties of the material.

Because Gd has paramagnetic properties, when it is close to the proton in resonance, it can effectively change the magnetic field of the proton and shorten the T_1_ and T_2_ relaxation times. In nuclear magnetic resonance imaging, Gd^3+^ can greatly increase imaging contrast and resolution. Jiaxi Ru [12] designed and synthesized a novel Gd(III) complex-based magnetic resonance imaging (MRI) contrast agent, GdL, which exhibited a high relaxivity (7.8 m/Ms), good water solubility (>100 mg mL^−1^), excellent thermodynamic stability, and high biosafety and biocompatibility. These results suggest that GdL is an excellent T1-weighted MRI contrast agent with potential applicability in clinical diagnosis. Additionally, Rugmani Meenambal [13] prepared multifunctional Gd^3+^, Dy^3+^ and Yb^3+^: β-Ca_3_(PO_4_)_2_ via a facile synthetic technique. The materials also displayed paramagnetic behaviour and exhibited high longitudinal (r1 = 48.71 m/Ms) and transverse relaxivity (r2 = 61.79 m/Ms), indicating their potential as T_1_ and T_2_ contrast agents in MRI. Furthermore, Alireza Shariati [14] developed and tested a new class of dual contrast agents based on polydopamine nanoparticles (PDA-NPs) that are functionalized and targeted with hyaluronic acid (HA). These nanoparticles (NPs) are chelated with Gd^3+^ to provide suitable contrast. The stability of the PDA/HA/Gd^3+^ material was evaluated by ICP–OES, zeta potentials, and DLS over 3 days, and the results suggested that the Gd-HA NPs were stable. If paramagnetic Gd^3+^ is doped into near-infrared-excited upconversion luminescent materials, high-precision imaging and accurate fluorescence temperature measurements can be achieved.

However, relatively few reports have investigated the doping mode of Gd^3+^ ions entering the lattice and their effects on material structural stiffness (Young’s modulus and Debye temperature), upconversion luminescence, temperature sensing, and magnetic properties. Bi_2_WO_6_ is a promising upconversion host because of its low phonon energy and stable physicochemical properties. However, its 2.7 eV band gap limits absorption to below 450 nm; thus, its upconversion luminescence has been explored primarily for photocatalysis. To broaden its spectral absorption and applications and to investigate its upconversion luminescence and temperature-sensing properties, on the basis of previous research on the fluorescence temperature measurement of Tm^3+^, Yb^3+^: Bi_2_WO_6_ [15], density functional theory was used to estimate the effects of different doping modes of Gd^3+^ on the lattice structure, band gap, and density of states of the material. The changes in Young’s modulus and Debye temperature of the material before and after Gd^3+^ doping were calculated. The effects of Gd^3+^ doping on the microstructure, upconversion luminescence, fluorescence thermometry, and magnetic properties of synthesized materials were analysed.

## 2. Results and Discussion

### 2.1. Structural Calculations

The Bi_2_WO_6_ host is part of the layered perovskite group called Bi_2_W_n_O_3n+3_ (n = 1). This structure includes layers that are positively charged (Bi_2_O_2_)^2+^ and layers arranged in octahedrons (WO_4_)^2−^. Defects in the (Bi_2_O_2_)^2+^ layer can interfere with the regular patterns and electronic properties of the crystal structure.

**Figure 1:**
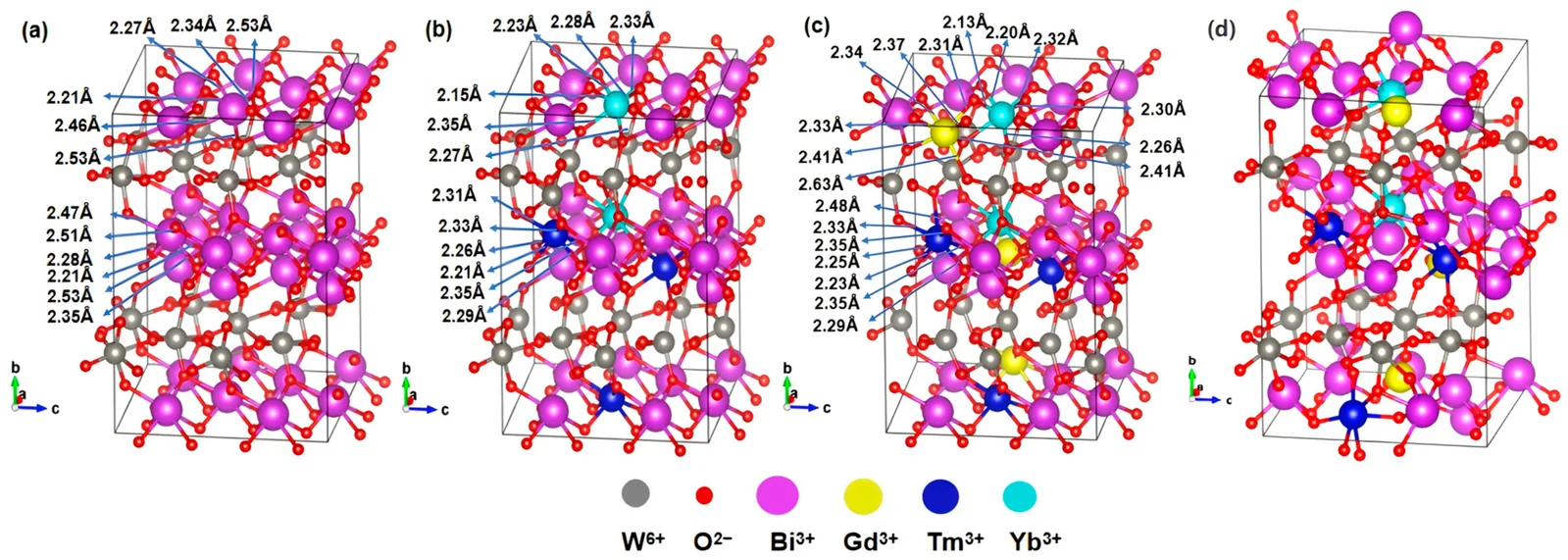
The crystal structure and bond angle of the material before and after doping (2 × 1 × 2 supercell): (**a**) Bi_2_WO_6_; (**b**)10% Tm^3+^, 6% Yb^3+^ substitution for Bi^3+^ sites; (**c**) 10% Tm^3+^, 6% Yb^3+^, 10% Gd^3+^ substitution for Bi^3+^ sites; (**d**) Tm^3+^ and Yb^3+^ substitution for Bi^3+^ sites and Gd^3+^ interstitial doping.

In the calculation, if the doping concentration of Tm^3+^ is 1% and the ion concentration is too low, the constructed supercell will be very large, and the result will be difficult to determine. To obtain effective calculation results, the Tm^3+^ doping concentration was set to 10%, and the calculated lattice structure and bond length around the doped Bi_2_WO_6_, Tm^3+^, Yb^3+^: Bi_2_WO_6_ and Tm^3+^, Yb^3+^, Gd^3+^: Bi_2_WO_6_ are shown in Figure 1.

To accurately describe the electronic structure of the system, Hubbard U was considered in the DFT calculations [16]. A value of U = 6.0 eV was introduced to the f-orbital of Tm, Gd and Yb. The formation energy of the supercells with different doping types was calculated using Equation (1).

(1) $$E_{f}=E_{\mathrm{dop}}-E_{\mathrm{perfect}}+\sum n_{i}E_{i}$$

where *E*_dop_ and *E_perfect_* are the total energies of the doped model and pristine supercells, respectively. n*_i_* and *E_i_* are the number and energy of the dopant species, respectively.

Table 1 shows the lattice parameters and formation energy calculated by VASP. When the small-radius ions Tm^3+^ (0.088 nm) and Yb^3+^ (0.0868 nm) undergo substitutional doping, the calculated unit cell volume decreases. When Tm^3+^, Yb^3+^, and Gd^3+^ (0.0938 nm) completely replace the large-radius Bi^3+^ (0.103 nm) ion lattice, the bond length around Tm^3+^/Yb^3+^/Gd^3+^ decreases, and the volume of the unit cell decreases further. When Tm^3+^/Yb^3+^ undergoes substitutional doping and Gd^3+^ undergoes interstitial doping, the calculated unit cell volume increases. The calculations reveal that the formation energies associated with Gd^3+^ substitutional doping and Gd^3+^ interstitial doping are −44.124 eV and −24.526, respectively. The negative values indicate that in both cases, the system energy decreases, suggesting good thermodynamic stability. Gd^3+^ substitutional doping results in a lower formation energy, leading to more stable compounds. The lower the formation energy is, the more stable the formed compound is.

**Figure 2:**
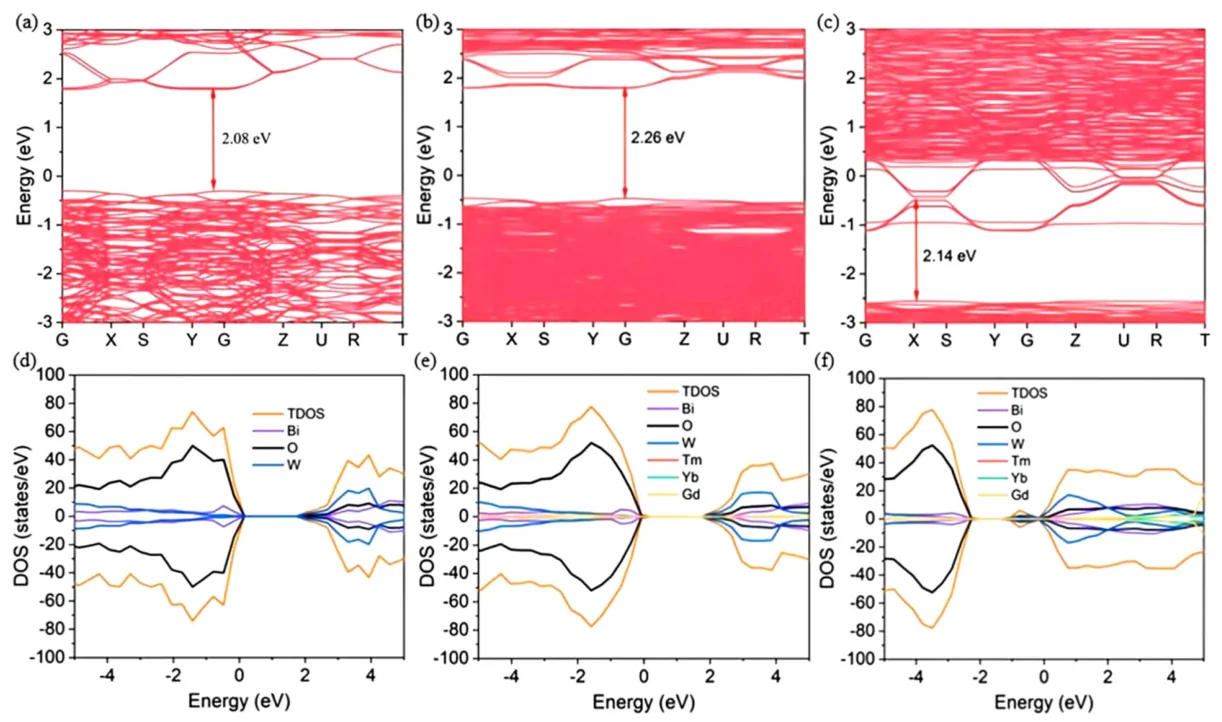
First-principles calculations of the band structure: (**a**) Bi_2_WO_6_; (**b**) 10% Tm^3+^, 6% Yb^3+^ and 10% Gd^3+^ substitutional doping; (**c**) 10% Tm^3+^ and 6% Yb^3+^ substitutional doping and 10% Gd^3+^ interstitial doping; (**d**–**f**) the corresponding electronic density of states.

Tm^3+^, Yb^3+^ and Gd^3+^ codoping result in Bi^3+^ in the matrix being replaced at multiple lattice sites, which can result in the formation of multiple structures. Considering the periodicity and symmetry of the crystal cell, the static energy of each structure was obtained by optimizing the atomic position. The structure with the lowest static energy was selected, and the energy band and density of states before and after Tm^3+^, Yb^3+^ and Gd^3+^ doping of Bi_2_WO_6_ were optimized. The band structures of the Bi_2_WO_6_ matrix, Tm^3+^/Yb^3+^/Gd^3+^-substituted doped samples, and Tm^3+^/Yb^3+^-substituted doped and Gd^3+^ gap-doped samples are shown in Figure 2a, Figure 2b, and Figure 2c, respectively. The corresponding electronic density of states maps are shown in Figure 2d–f. The energy band structure reveals that the calculated band gap of Bi_2_WO_6_ is 2.08 eV, which is lower than the experimental value of 2.69 eV. The reason is that the generalized gradient approximation functional cannot completely offset the Coulomb interaction between the electron and the material, resulting in excessive delocalization of the electron cloud, overestimation of the valence band top and underestimation of the conduction band bottom, resulting in a slightly smaller calculated band gap.

Compared with the Bi_2_WO_6_ matrix, when Tm^3+^/Yb^3+^/Gd^3+^ doping is performed to replace Bi^3+^, the calculated valence band top of the material shifts slightly downwards, and the band gap widens to 2.26 eV, as shown in Figure 2b. Owing to the weak hybridization between the 4f semi-full stable orbitals of Gd^3+^ and the O-2p valence band orbitals of the host lattice, the energy at the top of the valence band is slightly reduced. When Tm^3+^/Yb^3+^ doping is used to replace Bi^3+^ and Gd^3+^ is interstitially doped, impurity energy bands are calculated to appear between the band gaps. Both the conduction band and the valence band move synchronously towards lower energy, with a band gap of 2.14 eV, as shown in Figure 2c. Compared with Bi^3+^ (with an electronegativity of 1.2), Gd^3+^ ions have a higher electronegativity (1.9) and a higher effective ion potential in the trivalent state, resulting in a stronger ability to attract electrons. When the more electronegative Gd^3+^ is doped into the lattice, it strongly attracts the electron cloud of the surrounding O^2−^, reduces the overall average electrostatic potential of the system, and drives the conduction band and valence band to move downwards towards lower energies. When the doping concentration of the Gd^3+^ gap reaches the threshold, the 4f orbitals of adjacent Gd^3+^ overlap and broaden to form localized impurity bands that fill the band gap, providing intermediate energy bands for carrier transitions. Compared with the sample with Gd^3+^ substitutional doping (with a band gap of 2.26 eV), the sample with Gd^3+^ interstitial doping has a smaller band gap of 2.14 eV, which is beneficial for the matrix to absorb more low-energy photons.

### 2.2. Structural Stiffness

Typically, Young’s modulus *E* and Debye temperature *θ*_D_ are used to describe the structural stiffness of crystals [17]. Crystals with higher structural stiffness have weaker phonon energy, which limits electron–phonon coupling and leads to a lower probability of nonradiative relaxation [18]. The energy cutoff for plane-wave basis expansion was 400 eV, and the geometric structure relaxation convergence criterion was −0.02 eV/Å.

The elastic constants of the Bi_2_WO_6_ and Gd^3+^-doped Bi_2_WO_6_ matrices calculated using VASP are shown in Table 2. The stability was determined by measuring the elastic coefficients C_ij_ of the cubic crystalline phase Bi_2_WO_6_ as follows: C_11_ > 0, C_44_ > 0, C_55_ > 0, C_66_ > 0, C_11_C_22_ > C_12_^2^, and C_11_C_22_C_3_^3+^2C_12_C_13_C_23_-C_11_C_23_^2^-C_22_C_13_^2^-C_33_C_12_^2^ > 0. The calculated elastic coefficients *C*_ij_ of Gd^3+^-doped Bi_2_WO_6_ are shown in Table 2.

The Voigt and Reuss equations describe the upper and lower limits of the real crystal constants, respectively. The crystal moduli are taken as the Voigt–Reuss–Hill approximations, which are the arithmetic averages given by Voigt and Reuss. The calculation formulas are as follows:

(2) $$G=(G_{V}+G_{R})/2$$

(3) $$B=(B_{V}+B_{R})/2$$

Here, *G*_V_, which represents the shear modulus under the Voigt approximation, and *B*_V_, which represents the bulk modulus, can be expressed by the elastic coefficient *C*_ij_ of the Bi_2_WO_6_ crystal as follows:

(4) $$G_{V}=\frac{1}{15}[C_{11}+C_{22}+C_{33}+3(C_{44}+C_{55}+C_{66})-(C_{12}+C_{13}+C_{23})]$$

(5) $$B_{V}=\frac{1}{9}\left[C_{11}+C_{22}+C_{33}+2(C_{12}+C_{13}+C_{23})\right]$$

Here, the shear modulus *G*_R_ and bulk modulus *B*_R_ under the Reuss approximation have the following relationships with the elastic compliance coefficients *C*_ij_ of the crystal:

(6) $$\begin{array}{ll} G_{R} & =15\{4[C_{11}(C_{22}+C_{33}+C_{23})+C_{22}(C_{33}+C_{13})+C_{33}C_{12}\\ & -C_{12}(C_{23}+C_{12})-C_{13}(C_{12}+C_{13})-C_{23}(C_{13}+C_{23})]/\Delta \\ & +3[(1/C_{44})+(1/C_{55})+(1/C_{66})]{\}}^{-1} \end{array}$$

(7) $$\begin{array}{ll} B_{R} & =\Delta [C_{11}(C_{22}+C_{33}-2C_{23})+C_{22}(C_{33}-2C_{13})-2C_{33}C_{12}\\ & +C_{12}(2C_{23}-C_{12})]+C_{33}(2C_{12}-C_{13})+C_{23}(2C_{13}-C_{23}){]}^{-1} \end{array}$$

Here, Δ is formulated as follows:

(8) $$\Delta =C_{13}(C_{12}C_{23}-C_{13}C_{22})+C_{23}(C_{12}C_{13}-C_{23}C_{11})+C_{33}(C_{11}C_{22}-C_{12}^{2})$$

Young’s modulus *E* represents the structural stiffness of the material and is calculated as follows:

(9) E=9GB/(G+3B)

The compressional longitudinal wave velocity (*V*_P_) and transverse wave velocity (*V*_S_) are calculated as follows:

(10) $$V_{p}={\left[(\frac{4}{3}G+B)/\rho \right]}^{1/2}$$

(11) $$V_{S}=(G/\rho {)}^{1/2}$$

Here, *ρ* is the mass density of the matrix material.

The mean sound velocity *V*_m_ is calculated as follows:

(12) $$V_{m}={\left(\frac{2}{3V_{s}^{3}}+\frac{1}{3V_{p}^{3}}\right)}^{-\frac{1}{3}}$$

The Debye temperature *θ*_D_ can be obtained from the average sound speed and Debye approximation as follows:

(13) $$\theta_{D}=\frac{h}{k_{B}}{\left(\frac{3n}{4\pi V}\right)}^{\frac{1}{3}}V_{m}$$

where h is the Planck constant, k_B_ is the Boltzmann constant, *n* is the number of atoms in the primitive cell, *V* is the primitive cell volume, and *V*_m_ is the average phonon velocity.

The calculations show that Young’s modulus of the Bi_2_WO_6_ crystal is 184.210 GPa. When the same calculation method is used, Young’s modulus of the Bi_2_WO_6_ crystal doped with Gd^3+^ is 183.579 GPa, which is slightly smaller, as shown in Table 3.

Generally, the lower Young’s modulus is for a material, the weaker the interatomic forces, and materials with lower Young’s moduli undergo greater elastic deformation. After appropriate doping with Gd^3+^ ions, the matrix material is more prone to lattice distortion; this distortion reduces the lattice symmetry, which is beneficial for enhancing the luminescence.

Recent studies on perovskites have further revealed the correlation between structural rigidity and thermal stability, indicating that when the Debye temperature (quantifiable lattice vibration hardness) exceeds 200 K, nonradiative transitions can be effectively suppressed, resulting in stable luminescence [19]. In this work, through first-principles calculations, the Debye temperatures of the Bi_2_WO_6_ matrix and Gd^3+^-doped material were calculated to be 391.94 K and 392.45 K, respectively. These values are much higher than the above threshold, indicating excellent luminescence stability.

As shown in Table 4, according to the calculation results, compared with the NaYF_4_ host material, the Bi_2_WO_6_ host material and Gd^3+^-doped Bi_2_WO_6_ material have lower Debye temperatures. The lower the Debye temperature of the host is, the lower the upper limit of the lattice vibration frequency. Crossing the same electronic energy gap requires an increase in the number of phonons, which reduces the probability of nonradiative transitions. Importantly, Bi_2_WO_6_ also has a lower phonon energy, which is beneficial for improving the upconversion luminescence performance of the material.

### 2.3. Microstructural Characterization

**Figure 3:**
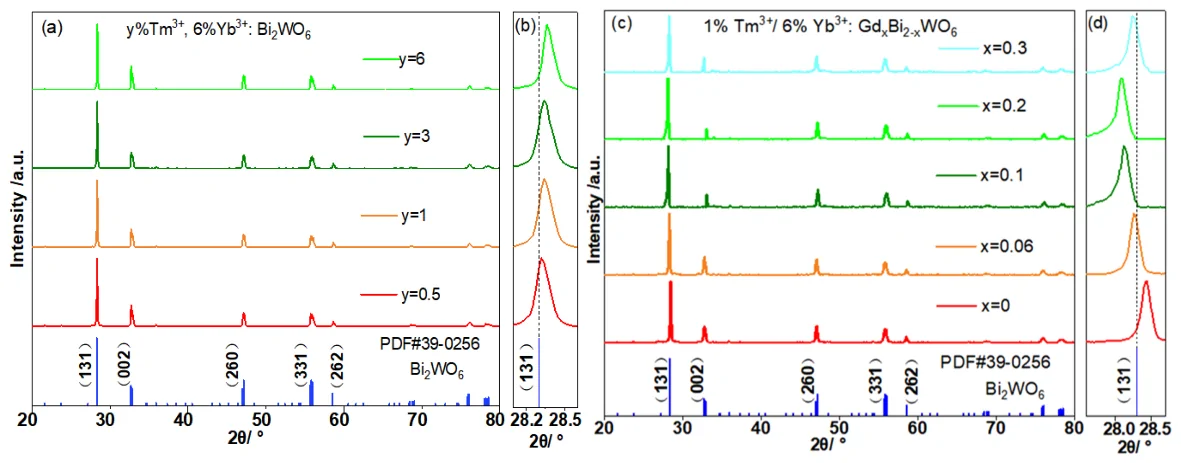
XRD patterns of the y% Tm^3+^/6% Yb^3+^: Gd_x_Bi_2−x_WO_6_ phosphors: (**a**) y = 0.5, 1, 3, 6; (**b**)The movement of the main diffraction peak (131) for (**a**); (**c**) x = 0, 0.06, 0.1, 0.2, 0.3; (**d**) The movement of the main diffraction peak (131) for (**c**).

The XRD patterns of the prepared y% Tm^3+^, 6% Yb^3+^: Gd_x_Bi_2__−__x_WO_6_ (x = 0, 0.06, 0.1, 0.2, 0.3; y = 0.5, 1, 3, 6) phosphors calcined at 800 °C for 3 h are shown in Figure 3. The diffraction peaks of all the prepared samples are essentially consistent with those of the Bi_2_WO_6_ standard card (PDF#39-0256). However, as the doping concentration of Tm^3+^ and Yb^3+^ ions increases, the diffraction peak shifts towards larger angles, as shown in Figure 3b. When smaller-radius Tm^3+^(0.088 nm)/Yb^3+^(0.0868 nm) ions replace Bi^3+^(0.103 nm), the cell volume decreases, the interplanar spacing d decreases, and the diffraction angle increases, resulting in the diffraction peaks shifting towards larger angles.

As the doping amount of low-concentration Gd^3+^ increases, the diffraction peak of the sample gradually shifts towards a smaller angle. When the amount of doped Gd^3+^ ions is x = 0.3, the diffraction peak (131) begins to reverse shift, as shown in Figure 3d. Although the formation energy of the sample with Gd^3+^ substitutional doping is lower, as shown in Table 1, when small-radius Gd^3+^ ions are doped into the lattice gap, the surrounding atoms spread, resulting in lattice expansion and a continuous increase in the interplanar spacing d. This is the core reason for the shift in the peak position towards small angles after Gd^3+^ doping. This finding also indicates that low-concentration doped Gd^3+^ ions are likely to enter the lattice gap. When the interstitial sites tend to saturate, excess Gd^3+^ ions are likely to replace the existing larger-radius Bi^3+^ ion sites in the lattice, resulting in cell shrinkage and a decrease in the interplanar spacing d. In addition, under high-concentration Gd^3+^ doping, the accumulated tensile strain of the lattice in the early stage reaches a critical value, and the lattice releases strain through local atomic rearrangement. The originally stretched interplanar spacing gradually decreases, resulting in a reverse shift in the diffraction peaks.

The JADE calculation results based on the XRD patterns are shown in Table 5, where a, b, and c are the cell parameters and V is the unit cell volume.

According to Scherrer’s formula [23], the grain size and the full width at half maximum (FWHM) of a diffraction peak are related as follows:

(14) $$D_{\mathrm{hkl}}=\frac{K\lambda }{\beta cos\;\theta }$$

where *D*_hkl_ is the grain size, K is Scherrer’s constant, λ is the wavelength of incident X-rays, β is the FWHM, and θ is the Bragg angle. K depends on the grain shape and size distribution, the diffraction line index, and the actual definition (FWHM or integration range). Assuming a constant *K*, the grain size of the samples with different Gd^3+^ doping concentrations was estimated as *D*_hkl_.

Compared with the original Bi_2_WO_6_ matrix, doping with appropriate amounts of Tm^3+^ and Yb^3+^ ions can lead to a decrease in the unit cell volume. As the concentration of Gd^3+^ ions gradually increases, the calculated unit cell volume first increases but then decreases. This finding indicates that when Gd^3+^ ions are doped into the lattice, interstitial doping is the main mode at low concentrations, whereas substitutional doping is the main mode after a certain concentration is reached. The VASP estimation results (Table 1) are consistent with the XRD experimental results (Figure 3) and JADE analysis results (Table 5) in terms of the trend of the unit cell volume change.

### 2.4. Morphological Analysis

**Figure 4:**
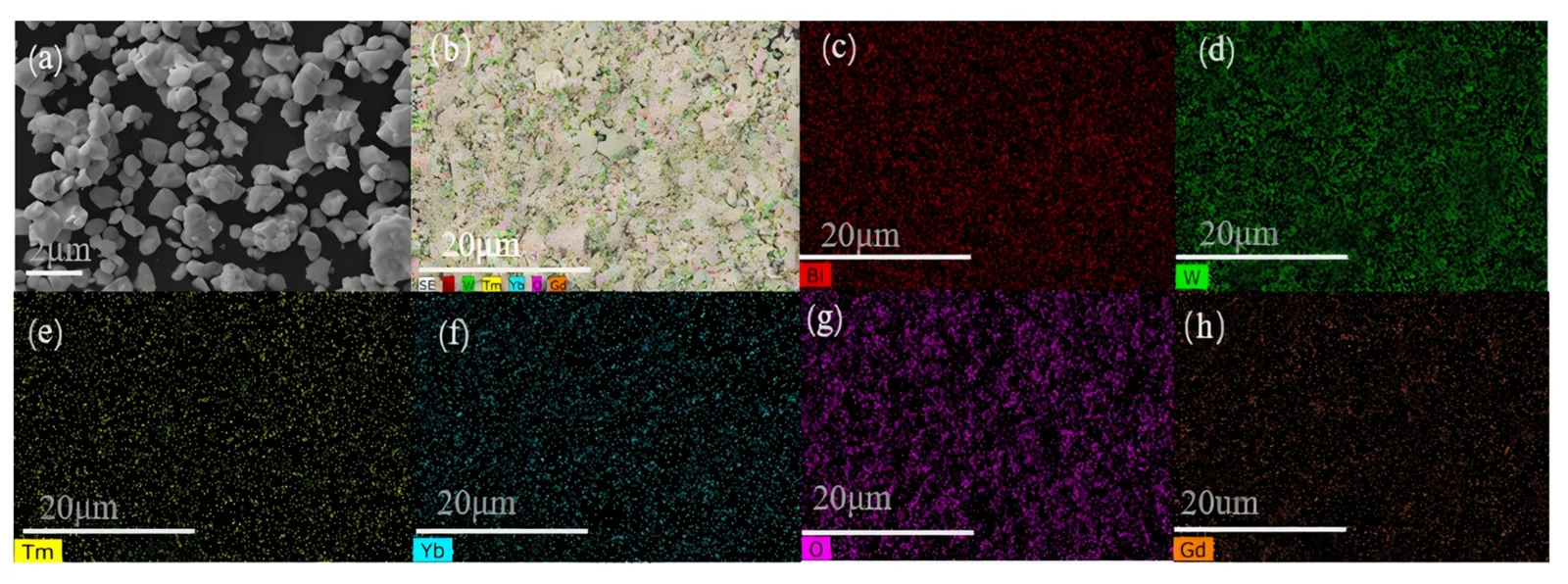
(**a**) SEM image of the 1% Tm^3+^, 6% Yb^3+^: Gd_0.2_Bi_1.8_WO_6_ sample; (**b**–**h**) SEM mapping of each element.

The scanning electron microscopy (SEM) image of the 1% Tm^3+^, 6% Yb^3+^: Gd_0.2_Bi_1.8_WO_6_ powder sample prepared by the high-temperature solid-phase method and calcined at 800 °C for 3 h is shown in Figure 4a. The SEM image reveals that the particle size is in the range of 0.5–3 μm, the particles have an inhomogeneous nearly spherical shape, the powder has undergone some agglomeration, and smaller particles are attached to the surface of larger particles. The SEM maps in Figure 4b–h show that Bi, W, O, Tm, Yb, and Gd are relatively uniformly distributed on the surface of the powder particles.

**Figure 5:**
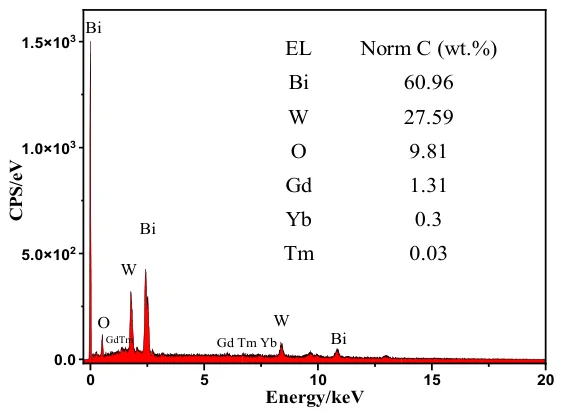
EDS energy spectrum of the 1% Tm^3+^, 6% Yb^3+^: Gd_0.2_Bi_1.8_WO_6_ sample.

The energy-dispersive X-ray spectroscopy (EDS) spectrum of the 1% Tm^3+^, 6% Yb^3+^: Gd_0.2_Bi_1.8_WO_6_ sample is shown in Figure 5. On the basis of the contents of Bi, W, O, Tm, Yb, and Gd, each element is uniformly distributed in the powder particles.

**Figure 6:**
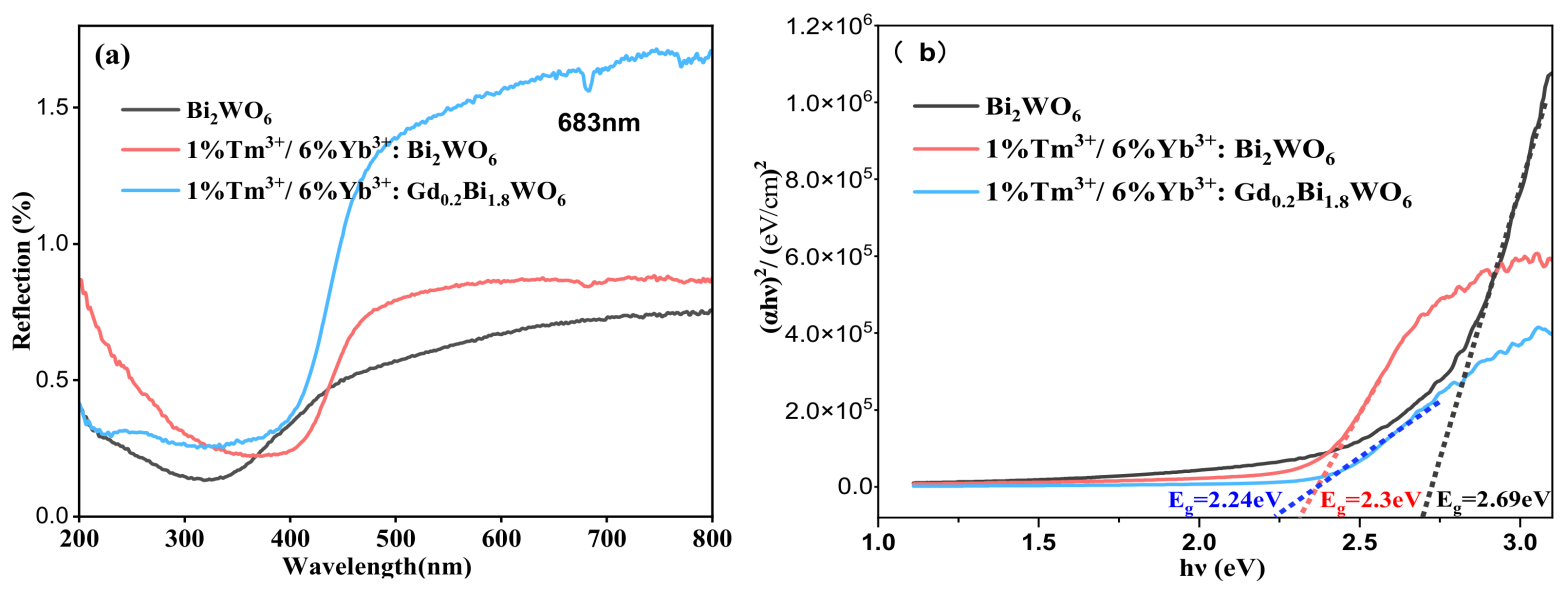
(**a**) Diffuse reflection spectra; (**b**) band gaps of Bi_2_WO_6_ and 1% Tm^3+^, 6% Yb^3+^: Gd_x_Bi_2−x_WO_6_ (x = 0, 0.2).

The UV–VIS diffuse reflection spectra of three samples, namely, the Bi_2_WO_6_ matrix, 1% Tm^3+^, 6% Yb^3+^: Bi_2_WO_6_ sample, and 1% Tm^3+^, 6% Yb^3+^: Gd_0.2_Bi_1.8_WO_6_ sample, are shown in Figure 6a. The samples containing Tm^3+^ have one weak absorption peak at 683 nm. To calculate the band gap (*E*_g_) of the three samples, the following formula was used [24]:

(15) $${\left[F\left(R\right)\mathrm{hv}\right]}^{1/n}=A\left(\mathrm{hv}\;-E_{g}\right)$$

(16) $$F(R)=(1-R{)}^{2}/2R$$

where *F*(*R*) is the absorption coefficient α and *R* is the reflection coefficient (direct semiconductor: n = 1/2; indirect semiconductor: n = 2). A is the absorption constant, *E*_g_ is the luminescence band gap, *ν* is the photon frequency, and *h* is the Planck constant.

The results in Figure 6a reveal that with Tm^3+^/Yb^3+^/Gd^3+^ doping, the diffuse reflectance spectra shift towards longer wavelengths, indicating that the material can absorb lower-energy long-wave photons. In addition, after codoping with Gd^3+^, the absorption peaks of Tm^3+^ around 683 nm and 779 nm became more pronounced. Figure 6b shows the band gap Eg before and after Tm^3+^/Yb^3+^/Gd^3+^ doping, which are 2.69 eV, 2.3 eV, and 2.24 eV, respectively. Compared with the Bi_2_WO_6_ matrix, the band gaps of the samples are reduced with codoping Tm^3+^/Yb^3+^/Gd^3+^, especially Gd^3+^ codoping. These results indicate that the impurity energy bands reduce the recombination probability of the electron–hole pairs, increase the probability of radiative transitions, and significantly increase the luminescence intensity.

The band gap obtained here deviates somewhat from the calculation results shown in Figure 2. The main reason for this is that the VAPS calculations did not consider the actual probabilities of different Bi^3+^ sites and interstitial sites being occupied after doping. This may change the symmetry of the computational structure, and irreducible high-symmetry points may be offset relative to undoped points.

### 2.5. Upconversion Luminescence Properties

**Figure 7:**
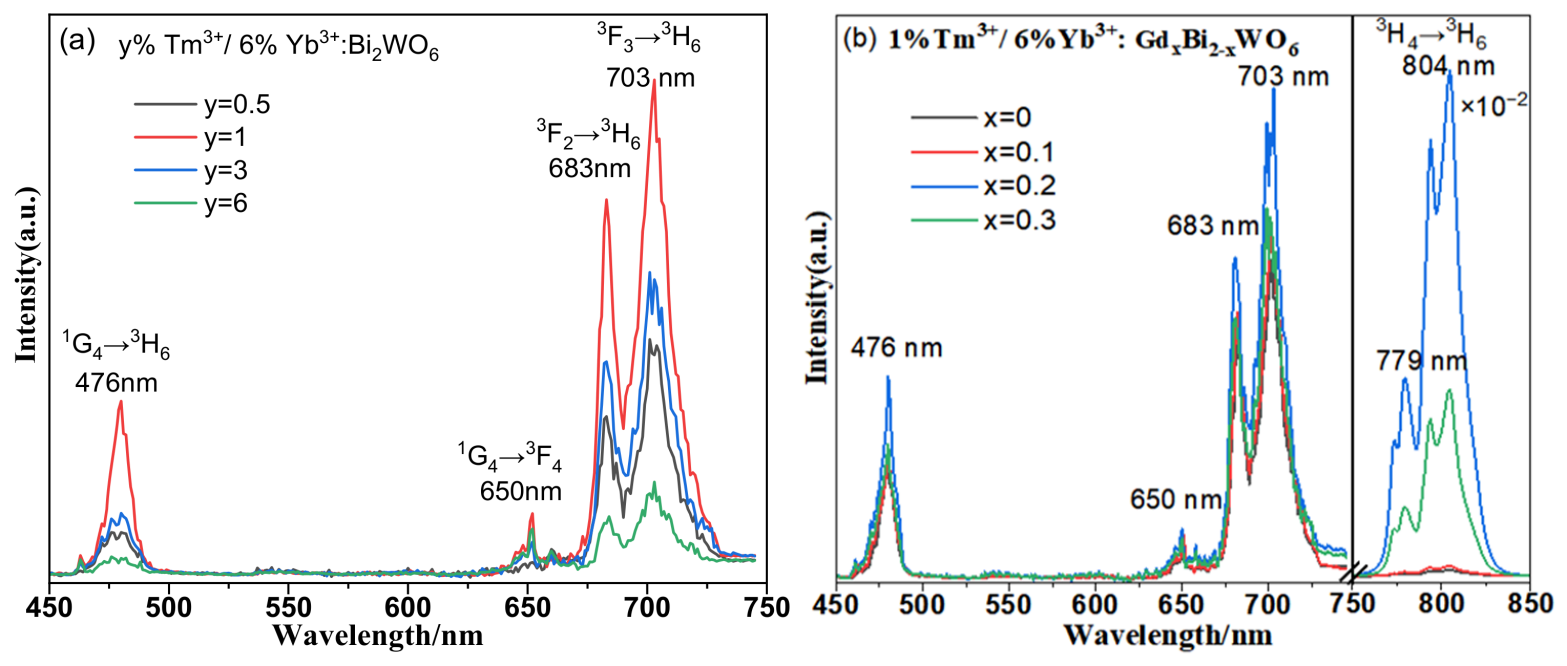
Emission spectra of the y% Tm^3+^, 6% Yb^3+^: Gd_x_Bi_2−x_WO_6_ samples under 980 nm laser excitation: (**a**) y = 0.5, 1, 3, 6; (**b**) x = 0, 0.1, 0.2, 0.3.

The spectra of y% Tm^3+^, 6% Yb^3+^: Gd_x_Bi_2−x_WO_6_ (x = 0, 0.1, 0.2, 0.3; y = 0.5, 1, 3, 6) under 980 nm laser excitation are shown in Figure 7. At room temperature, under 980 nm excitation, all samples exhibited characteristic peaks of Tm^3+^, such as those at 476 nm (^1^G_4_→^3^H_6_), 650 nm (^1^G_4_→^3^F_4_), 683 nm (^3^F_2_→^3^H_6_), 703 nm (^3^F_3_→^3^H_6_), 779 nm (^3^H_4(1)_→^3^H_6_) and 804 nm (^3^H_4(2)_→^3^H_6_).

When the doping concentrations of Yb^3+^/Tm^3+^ ions are 6% and 1%, respectively, and the doping amount of Gd^3+^ is x = 0.2, the synthesized Tm^3+^, Yb^3+^: Gd_0.2_Bi_1.8_WO_6_ sample exhibits the strongest luminescence, as shown in Figure 7a,b. Compared with that of the sample without Gd^3+^doping, when the Gd^3+^ doping amount is x = 0.2, the emission intensities of Tm^3+^ at 476 nm, 683 nm, 703 nm, and 804 nm increase by 1.8, 1.24, 1.35, and 81.9 times, respectively, as shown in Figure 7b. Many factors affect the luminescence of synthetic materials. The main reasons that Gd^3+^ doping increases the luminescence of Tm^3+^ materials are as follows.

Gd^3+^ and Bi^3+^ have the same valence, and substitution does not introduce intrinsic charge defects (such as vacancies), but size mismatches during ion doping may lead to lattice dislocations or oxygen vacancies to alleviate strain. Dislocations typically reduce luminescence (as nonradiative recombination centres), whereas moderate oxygen vacancies can increase luminescence. If Gd^3+^ doping occurs in the lattice interstitial state, the Bi–O bond within the lattice will be weakened or even broken, resulting in a small amount of oxygen atoms leaving the lattice and forming new oxygen vacancies. The newly added oxygen vacancies can serve as electron capture centres, introducing intermediate energy levels in the band gap, reducing the energy required for electron transitions, increasing the number of particles in the excited state, and increasing the probability of radiative transitions. The above results are consistent with the relevant content reported in the literature [25,26].

The introduction of an appropriate amount of Gd^3+^ can disperse the luminescent centre, namely, Tm^3+^, thereby avoiding the nonradiative dissipation caused by cross-energy transfer due to Tm^3+^ ion aggregation and allowing more energy to be released in the form of radiative luminescence. In addition, if Gd^3+^ with a smaller radius replaces Bi^3+^ with a larger radius, the adjacent M–O (M = Gd/Bi) bond length decreases, the local lattice shrinks, and compressive strain increases. At this point, owing to the increased probability of valence electron orbital overlap, the band gap of the material decreases. During the same excitation process, more electrons are excited, thereby promoting the upconversion process. Gd^3+^ doping does not change the main crystal structure of bismuth tungstate but does reduce the band gap, causing a redshift in the absorption edge of the material, which can then capture more long-wavelength photons and provide more energy for the upconversion luminescence process, thereby increasing the luminescence.

In the layered structure of perovskite, doped Gd^3+^ can adapt to irregular sites, especially at high doping concentrations, resulting in the formation of local disorder, increasing the probability of nonradiative recombination, increasing the number of defect states, and decreasing the luminescence.

On the basis of the energy level positions of Tm^3+^ in Gd_x_Bi_2−x_WO_6_ (^3^H_4_: 5800 cm^−1^, ^3^F_4_: 2900 cm^−1^), the energy mismatch during this process (^3^H_4_ +^3^H_6_→^3^F_4_+ ^3^F_4_ (CR_1_)) is close to zero, making it highly resonant and allowing it to be assisted by a single phonon [27]. Gd^3+^ doping tunes this rate by altering the lattice constant and Tm^3+^–Tm^3+^ distance. As the ion concentration increases, the CR_1_ energy transfer efficiency increases, resulting in reduced luminescence. In accordance with the literature [28], in the Tm^3+^-doped system, ^1^G_4_ + ^3^H_6_ → ^3^H_4_ + ^3^H_5_ (CR_2_) and ^1^G_4_ + ^3^H_6_→^3^H_5_ + ^3^H_4_ (CR_3_) are identified as the primary cross-relaxation pathways for the ^1^G_4_ energy level. Moreover, the reduced matrix elements (Uλ)^2^ of these two processes are relatively large, ensuring a high energy transfer rate. These channels have also been reported in Gd_2_O_2_S: Tm^3+^ [29]. At excessively high concentrations of Gd^3+^ ions, the ion spacing rapidly decreases, and the cross-energy transfer (^3^H_4_+^3^H_6_→^3^F_4_+^3^F_4_, ^1^G_4_+^3^H_6_→^3^H_5_+^3^H_4_, ^1^G_4_+^3^H_6_→^3^H_4_+^3^H_5_) between ions and the probability of nonradiative transitions increase; thus, the luminescence decreases.

**Figure 8:**
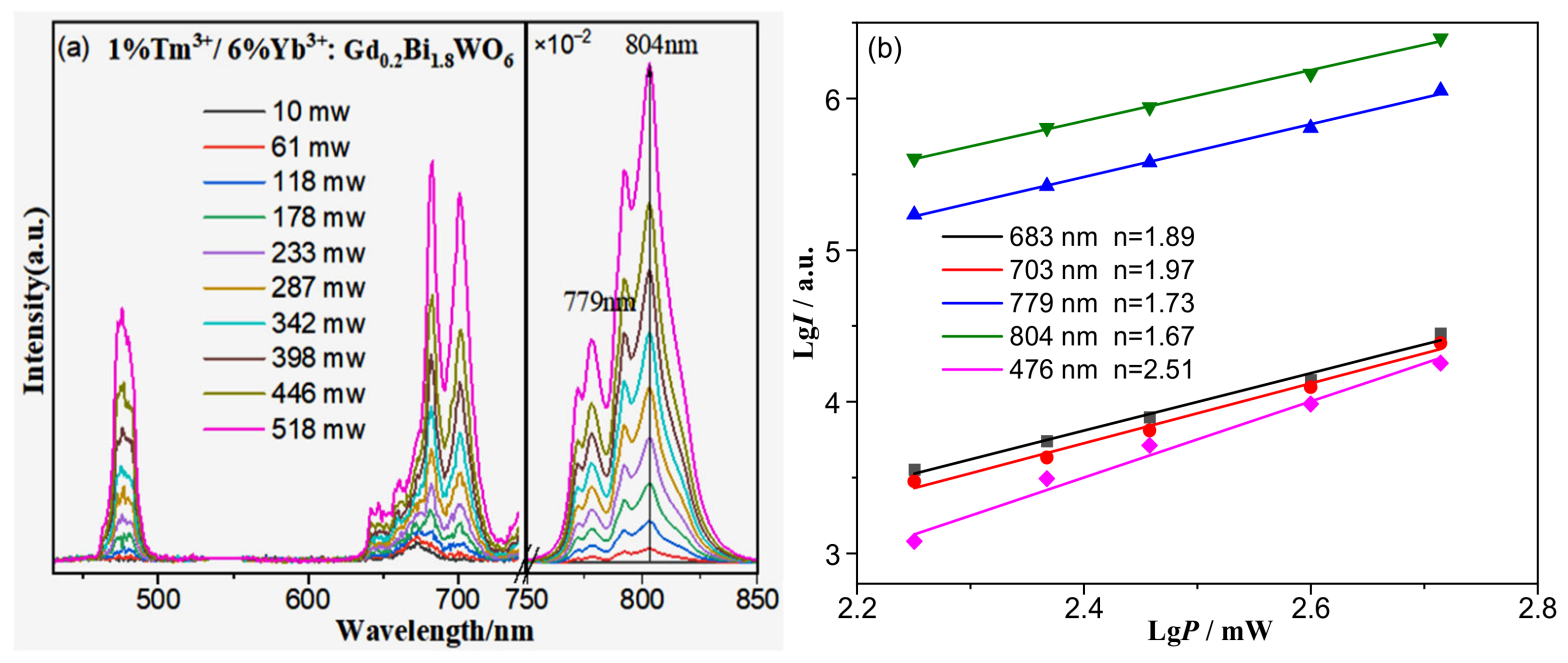
The 1% Tm^3+^, 6% Yb^3+^: Gd_0.2_Bi_1.8_WO_6_ sample: (**a**) Relationship between the luminescence intensity and the excitation power; (**b**) Linear fitting of Lg*I* and Lg*P.*

The upconversion luminescence spectra of the 1% Tm^3+^, 6% Yb^3+^: Gd_0.2_Bi_1.8_WO_6_ sample measured under 980 nm laser excitation at powers ranging from 10 mW to 518 mW are shown in Figure 8a. All the intensities *I* of the 476 nm, 683 nm, 703 nm, and 804 nm emission peaks of Tm^3+^ in the sample gradually increase with increasing power *P.* According to the literature [30], the number of absorbed photons n can be determined on the basis of *I*∝$P^{\;n\;}$. The value of n is influenced by the competition between the nonradiative relaxation probability and the radiative transition probability of intermediate ions.

The logarithmic relationships between the luminescence intensities *I* at 476 nm, 683 nm, 703 nm, 779 nm and 804 nm and the power *P* were fitted (178–518 mW), as shown in Figure 8b. The numbers of absorbed photons n corresponding to these emission peaks of Tm^3+^ are calculated to be 2.51, 1.89 1.97, 1.73 and 1.67, indicating that the peaks at 683, 703, 779 and 804 nm originate from two-photon absorption and that the peak at 476 nm originates from three-photon absorption.

When the excitation pump power increases, the probability of Yb^3+^ ions being excited increases, the probability of energy transfer from Yb^3+^ ions to Tm^3+^ ions increases, the probability of radiative transitions of Tm^3+^ ions increases, and the luminescence of the sample increases. When the excitation power increases to 518 mW, the thermal effect caused by the high power increases the probability of cross-relaxation between ions and the nonradiative relaxation probability, saturation effects occur, and the luminescence intensity *I* and power *P* have a nonlinear relationship. Thus, the number of absorbed photons n cannot be determined. Therefore, the best power of 518 mW was selected for this experiment.

**Figure 9:**
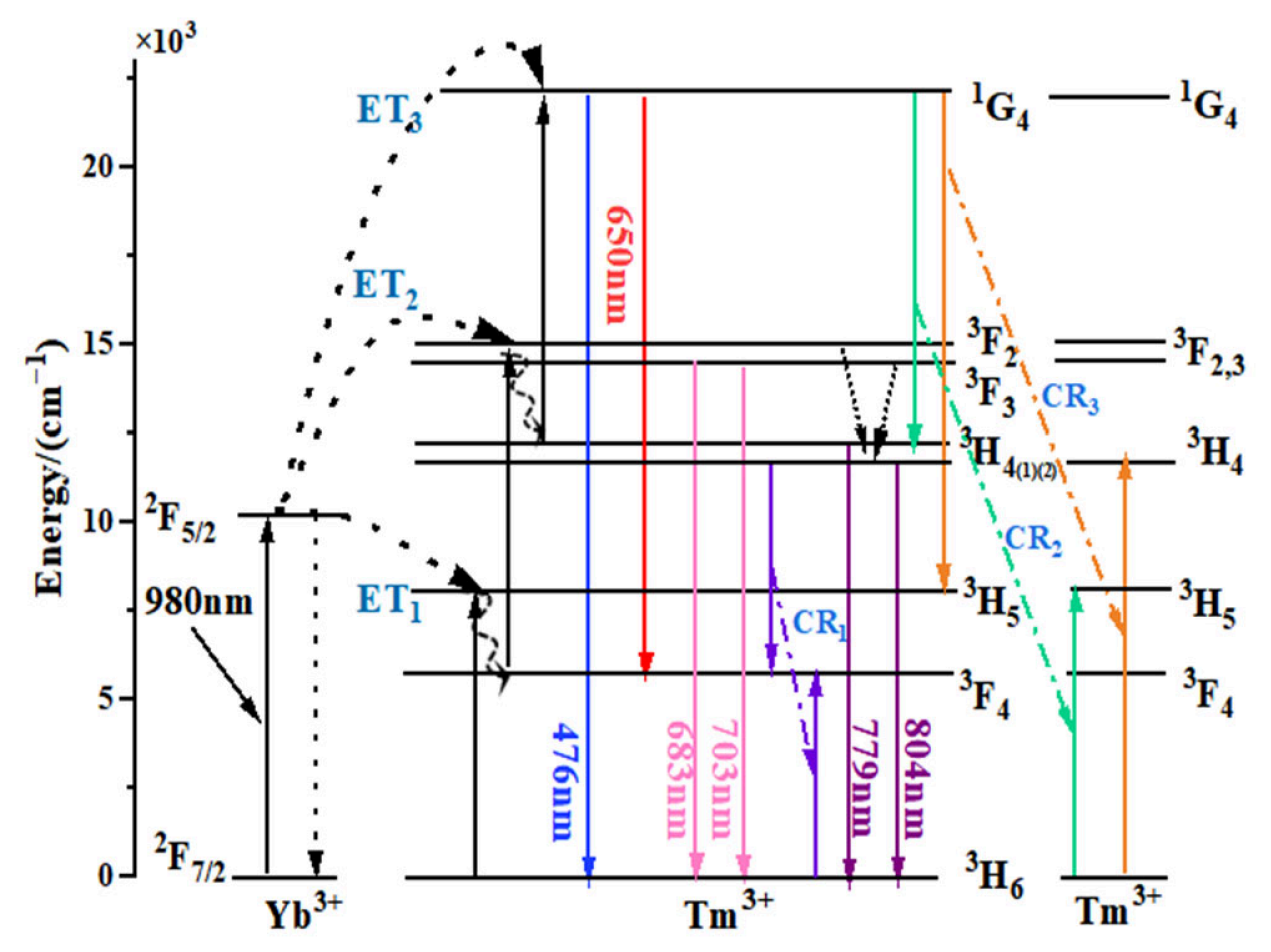
Energy transfer and energy level transition of Yb^3+^ and Tm^3+^ under 980 nm laser excitation.

The energy transfer and energy level transition of Yb^3+^ and Tm^3+^ under 980 nm laser excitation are shown in Figure 9. The Tm^3+^ ions in the ground state ^3^H_6_ absorb some of the energy and transition to ^3^H_5_. Yb^3+^ has a large absorption cross-section at 980 nm, so Yb^3+^ absorbs photons under 980 nm excitation and transitions to the excited state ^2^F_5/2_. Because the ^2^F_5/2_ energy level of Yb^3+^ and the ^3^H_5_ energy level of Tm^3+^ do not exactly match, an energy transfer process from Yb^3+^ to Tm^3+^ occurs with the assistance of phonons. ET_1_, ET_2_ and ET_3_ in the figure represent the transfer of energy from Yb^3+^ to Tm^3+^ [15]. Tm^3+^ ions at ^3^H_5_ undergo nonradiative relaxation to ^3^F_4_, and Tm^3+^ ions accept energy from Yb^3+^ and transition from ^3^F_4_ to ^3^F_2,3_. Some Tm^3+^ ions in the excited state level ^3^F_2,3_ transition to the ground state ^3^H_6_ and emit red light at 683 and 703 nm. Some Tm^3+^ ions at the ^3^F_2,3_ level undergo nonradiative relaxation to the splitting energy level ^3^H_4(1)(2)_ state. Here, ^3^H_4_ is a multienergy level that is split into upper and lower energy levels, denoted ^3^H_4(1)_ and ^3^H_4(2)_. When Tm^3+^ ions transition from ^3^H_4(1)(2)_ to ^3^H_6_, they produce near-infrared light with wavelengths of 779 nm and 804 nm. With the help of phonons, the Yb^3+^ ions in the excited state of ^2^F_5/2_ transfer energy to the Tm^3+^ ions at the ^3^H_4_ level, and the Tm^3+^ ions are excited from ^3^H_4_ to ^1^G_4_. Some Tm^3+^ ions at the ^1^G_4_ energy level transition to the ground state ^3^H_6_ and emit blue light at 476 nm, and a small number of Tm^3+^ ions at the ^1^G_4_ energy level transition to the ^3^F_4_ energy level and emit red light at 650 nm [15].

**Figure 10:**
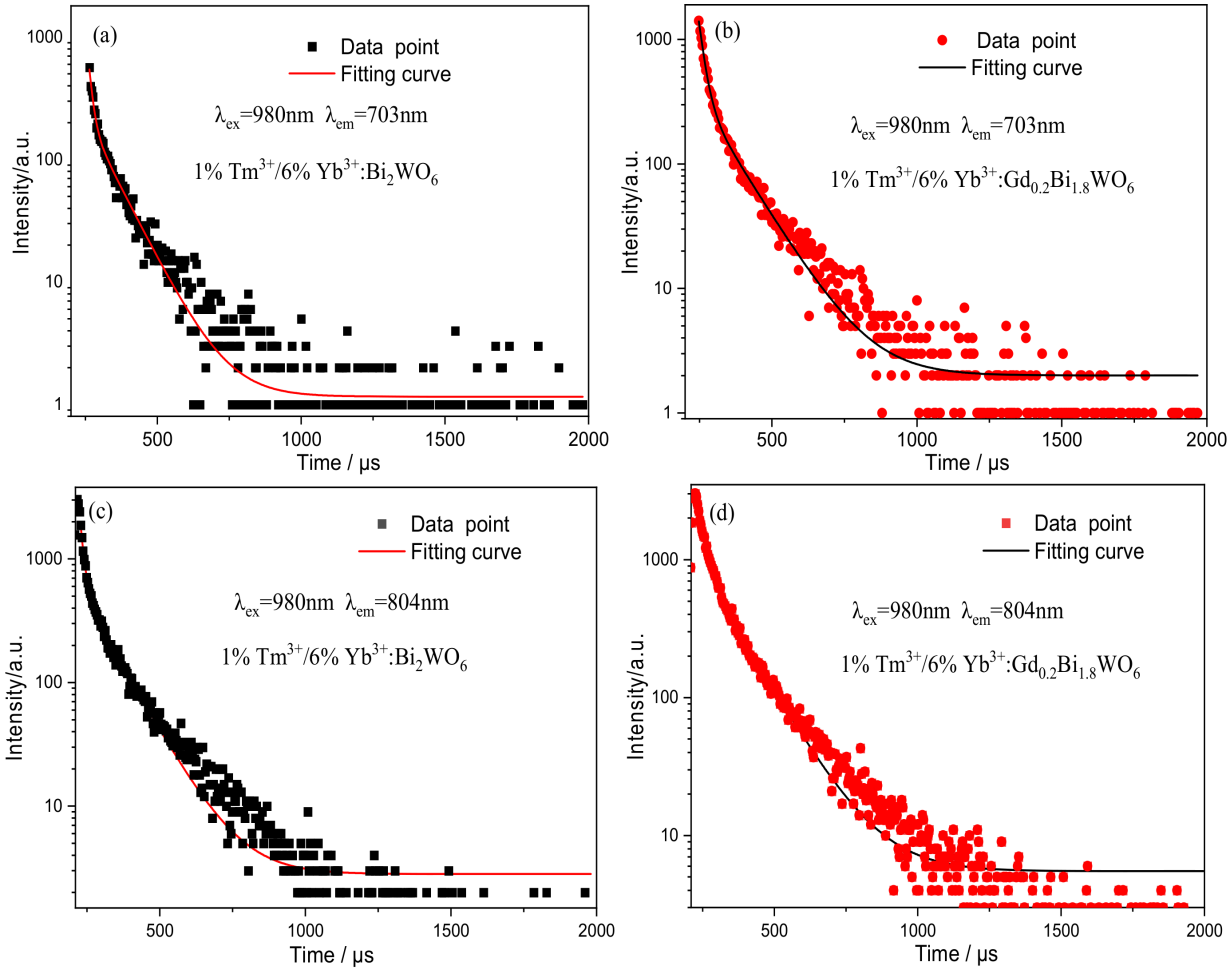
Energy level decay curves and fitting curves of the 1% Tm^3+^, 6% Yb^3+^: Gd_x_Bi_2−x_WO_6_ samples: (**a**) x = 0, λ_ex_ = 703 nm; (**b**) x = 0.2, λ_ex_ = 703 nm; (**c**) x = 0, λ_ex_ = 804 nm; (**d**) x = 0.2, λ_ex_ = 804 nm.

The energy level decay curves of ^3^F_3_→^3^H_6_ and ^3^H_4(2)_→^3^H_6_ under 980 nm excitation for the 1% Tm^3+^, 6% Yb^3+^: Gd_x_Bi_2−x_WO_6_ (x = 0, 0.2) samples are shown in Figure 10. By fitting the energy level decay curve with a double exponential function, the energy level lifetime τ is calculated as follows:

(17) $$I(t)=A_{1}e^{-t/\tau 1}+A_{2}e^{-t/\tau 2}$$

(18) $$\tau =\frac{A_{1}{\tau_{1}}^{2}+A_{2}{\tau_{2}}^{2}}{A_{1}\tau_{1}+A_{1}\tau_{2}}$$

where *I*(t) is the luminescence intensity and *A*_1_ and *A*_2_ are coefficients. τ_1_ and τ_2_ are the radiative transition lifetime and radiation-free relaxation lifetime, respectively. τ is the energy level lifetime.

The lifetimes of the ^3^F_3_ and ^3^H_4(2)_ energy levels of the sample before and after Gd^3+^ doping are shown in Table 6. After the material was doped with Gd^3+^, the lifetimes of the ^3^F_3_ and ^3^H_4(2)_ energy levels for Tm^3+^ in the synthesized material increased to varying degrees. The analysis results in Figure 6 show that moderate codoping with Gd^3+^ shifted the absorption band of the material towards longer wavelengths. These results are consistent with the predicted trend shown in Figure 2b,c, indicating that Gd^3+^ doping narrows the matrix band gap, which is beneficial for the matrix to absorb more low-energy photons and promote energy upconversion. Therefore, Gd^3+^ codoping can optimize the energy transfer from Yb^3+^ to Tm^3+^ through energy exchange interactions, thereby extending the lifetime of the Tm^3+^ energy levels.

### 2.6. Temperature Sensing Characteristics

**Figure 11:**
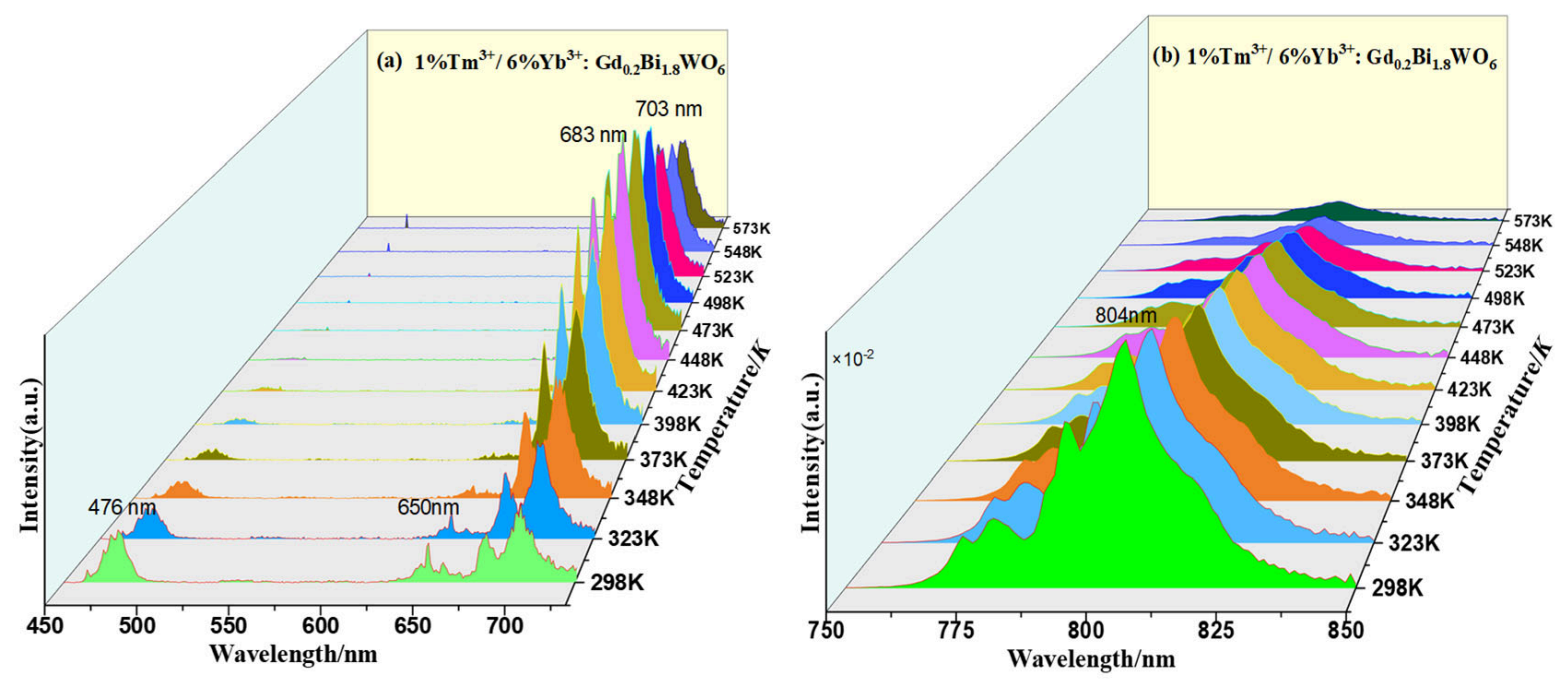
Emission spectra of the 1% Tm^3+^, 6% Yb^3+^: Gd_0.2_Bi_1.8_WO_6_ sample at 298~573 K: (**a**) 450–750 nm, (**b**) 750–850 nm.

The upconversion emission spectra of the 1% Tm^3+^, 6% Yb^3+^: Gd_0.2_Bi_1.8_WO_6_ sample in the range of 450–750 nm under 980 nm laser excitation (518 mW) and variable temperature conditions (298–573 K) are shown in Figure 11a. The upconversion emission spectra from 750–850 nm at 298 to –573 K under the same excitation conditions is shown in Figure 11b.

The intensities of the 804 nm, 650 nm and 476 nm emission peaks monotonically decrease as the temperature increases from 298 K to 573 K, whereas those of the 703 nm and 683 nm emission peaks first increase but then decrease with increasing temperature. Owing to the temperature sensitivity of the thermal coupling energy levels ^3^F_2,3_ and ^3^H_4_, with the assistance of phonons, the number of particles thermally excited at the ^3^F_2,3_ energy levels begins to increase, which leads to an increase in the probability of radiative transitions from the ^3^F_2,3_ energy levels and increases the emission intensities at 683 nm and 703 nm, corresponding to the thermal coupling energy levels. Moreover, the probability of the particles in the ^3^F_2,3_ energy level relaxing to the ^3^H_4_ energy level decreases. Therefore, the probabilities of emissions from the ^3^H_4_ transition to ^3^H_6_ and from the ^3^F_2,3_ excited state to ^1^G_4_ decrease, and the luminescence intensities at 804 nm, 779 nm, 650 nm and 476 nm decrease.

The relationship between the fluorescence intensity ratio (*FIR*) and temperature based on the thermal coupling energy levels of luminescent materials follows the Boltzmann distribution function [30]:

(19) $$\mathrm{FIR}=\frac{I_{1}}{I_{2}}=A\exp\left(-\frac{\Delta E}{\;k_{B}T}\right)$$

where *I*_1_ and *I*_2_ denote the fluorescence intensities of the two peaks from the thermal coupling energy levels; *FIR* is the ratio of the intensities of the two peaks; and A is a constant related to the energy level simplicity, spontaneous radiation rate, and phonon energy of Tm^3+^. ∆*E* is the energy level spacing between the upper thermal coupling energy levels, and k_B_ is the Boltzmann constant.

The thermal coupling energy levels ^3^F_3_ and ^3^H_4_ transition to the ^3^H_6_ energy level, emitting light at 703 nm and 804 nm, respectively. The fluorescence intensity ratio at 703 nm and 804 nm varies with temperature *T* as follows:

(20) $$\mathrm{FIR}=\frac{I_{703\mathrm{nm}}}{I_{804\mathrm{nm}}}=0.3396\exp(-1807.4475/T)$$

**Figure 12:**
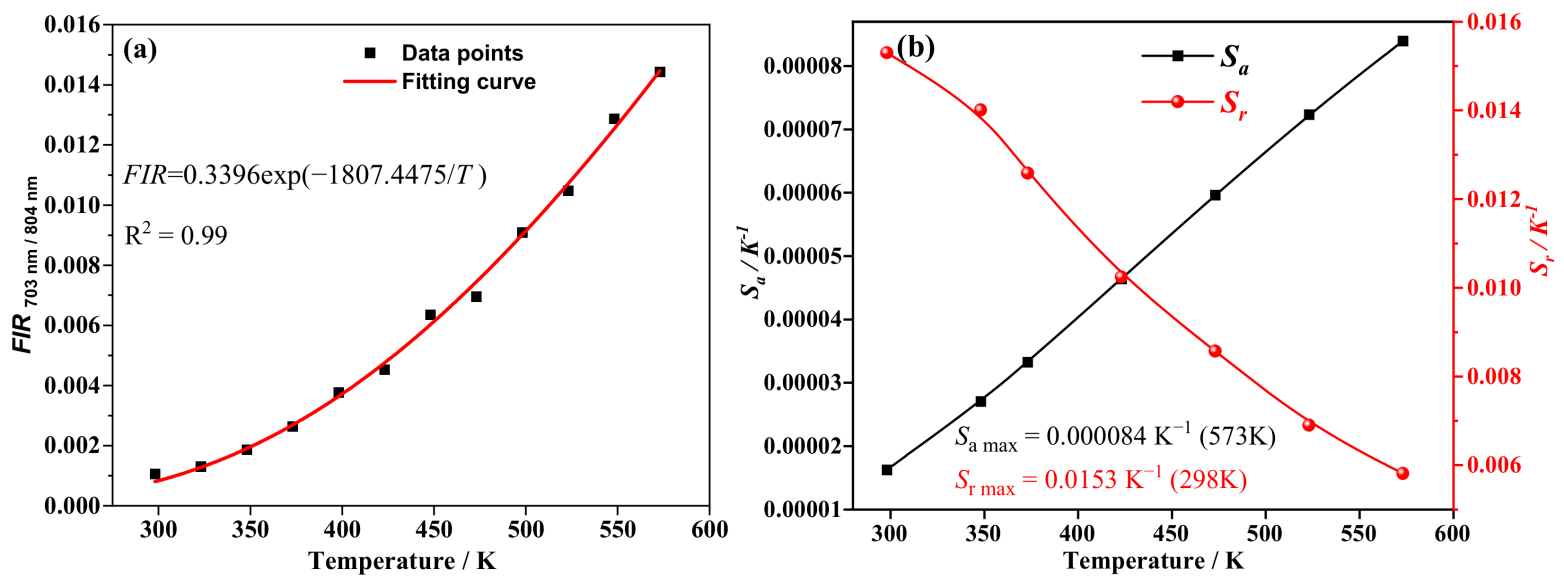
(**a**) Fluorescence intensity ratio of the 703 nm and 804 nm peaks versus temperature. (**b**) *S_a_* and *S_r_.*

The relationship between *FIR*_703nm and 804nm_ and temperature was fitted, as shown in Figure 12a.

In Figure 12a, R^2^ indicates the degree of agreement between the theoretical fit and the experimental data, and the theoretical values are in perfect agreement with the experimental values when R^2^ = 1.

In accordance with the literature [31], the absolute thermometry sensitivity *S_a_* can be expressed as follows:

(21) $$S_{a}=|\frac{dFIR}{dT}|$$

The relative thermometry sensitivity *S_r_* can be expressed as follows:

(22) $$S_{r}=|\frac{1}{FIR}\frac{dFIR}{dT}|$$

The relationships of the absolute thermometry sensitivity *S*_a_ and relative thermometry sensitivity *S_r_* with temperature were calculated for the 1% Tm^3+^, 6% Yb^3+^: Gd_0.2_Bi_1.8_WO_6_ sample at temperatures ranging from 298 to 573 K, as shown in Figure 12b. The obtained *S_r_* of the material decreases with increasing temperature. At 298 K, the maximum *S*_a_ and *S_r_* values are 0.000084 K^−1^ (573 K) and 0.0153 K^−1^ (298 K), respectively.

The fluorescence intensity ratio for the nonthermal coupling energy levels can be used to measure the temperature; according to reference [32], a polynomial function can be used to establish a quantitative relationship between *FIR* and temperature, and the equation is as follows:

(23) $$FIR=A+BT+CT^{2}+DT^{3}$$

where *FIR* is the intensity ratio of the two luminescence peaks; *T* denotes the absolute temperature; and *A*, *B*, *C* and *D* are undetermined coefficients.

**Figure 13:**
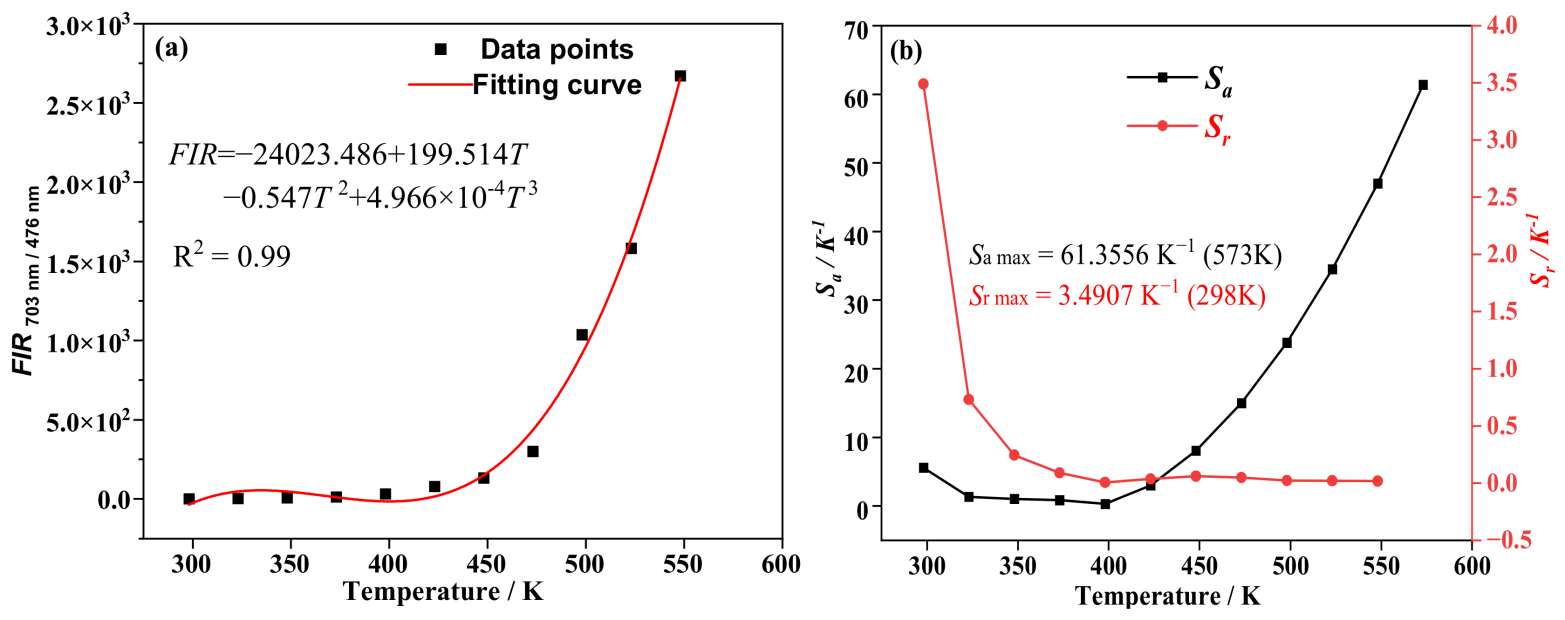
(**a**) Fluorescence intensity ratio of the 703 nm and 476 nm peaks versus temperature; (**b**) *S_a_* and *S_r_.*

The *FIR* values of the 703 nm and 476 nm peaks (nonthermal coupling energy level pairs ^3^F_3_/^1^G_4_→^3^H_6_) versus temperature for this sample are shown in Figure 13a. The fitted *FIR* versus *T* relationship is as follows:

(24) $$\mathrm{FIR}=\frac{I_{703\mathrm{nm}}}{I_{476\mathrm{nm}}}=-24023.486+199.514T-0.547T^{\;2}+4.966\times {10^{-4}T}^{\;3}$$

On the basis of *FIR*_703nm/476nm_, *S*_a_ and *S_r_* were calculated from 298 to 573 K, as shown in Figure 13b. The maximum *S_a_* is 61.3556 K^−1^ (573 K), and the maximum *S_r_* is 3.4907 K^−1^ (298 K).

The temperature sensing properties of rare earth ions and Gd^3+^-codoped upconversion materials with different matrix materials are shown in Table 7. Under 980 nm excitation, the 1% Tm^3+^, 6% Yb^3+^: Gd_0.2_Bi_1.8_WO_6_ sample prepared in this work exhibits better properties compared with the materials reported in the literature [7,8,33,34,35] in terms of the maximum *S_r_*. Compared with that of the undoped Gd^3+^ sample [15], the maximum *S_r_* of the Gd^3+^-codoped sample was 92.35 times larger.

The temperature resolution *δ*_T_ refers to the smallest change in temperature that a temperature-sensing material can sense [36] and is calculated as follows:

(25) $$\delta_{T}=\frac{1}{S_{r}}\frac{\delta \Delta }{\Delta }$$

(26) $$\frac{\delta \Delta }{\Delta }=\sqrt{{\left(\frac{\delta I}{I_{1}}\right)}^{2}+{\left(\frac{\delta I}{I_{2}}\right)}^{2}}$$

where δΔ/Δ represents the relative uncertainty in the fluorescence intensity ratio, *S_r_* is the relative thermometry sensitivity, Δ is the fluorescence intensity ratio, *δI* is the uncertainty in the fluorescence intensity, and *I_1_* and *I_2_* are the intensities of the two fluorescence peaks.

**Figure 14:**
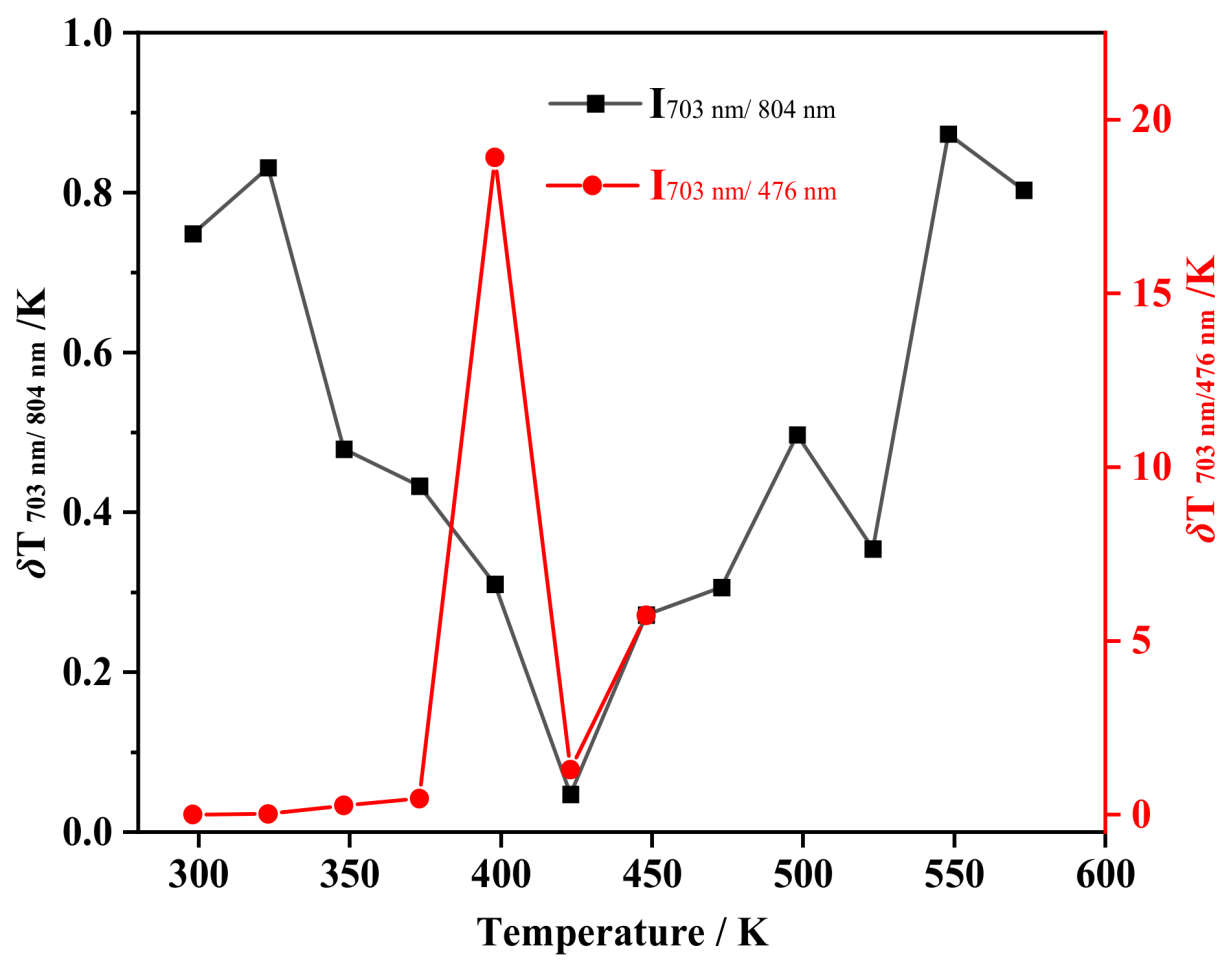
Temperature resolution $\delta_{T}$ at different temperatures for 1% Tm^3+^, 6% Yb^3+^: Gd_0.2_Bi_1.8_WO_6_.

The $\delta_{T}$ of this sample based on the *FIR*_703nm/804nm_ or *FIR*_703nm/476nm_ value was calculated according to Equations (25) and (26), as shown in Figure 14. The corresponding minimum $\delta_{T}$ values are 0.0474 K at 423 K and 0.0083 K at 298 K, respectively.

**Figure 15:**
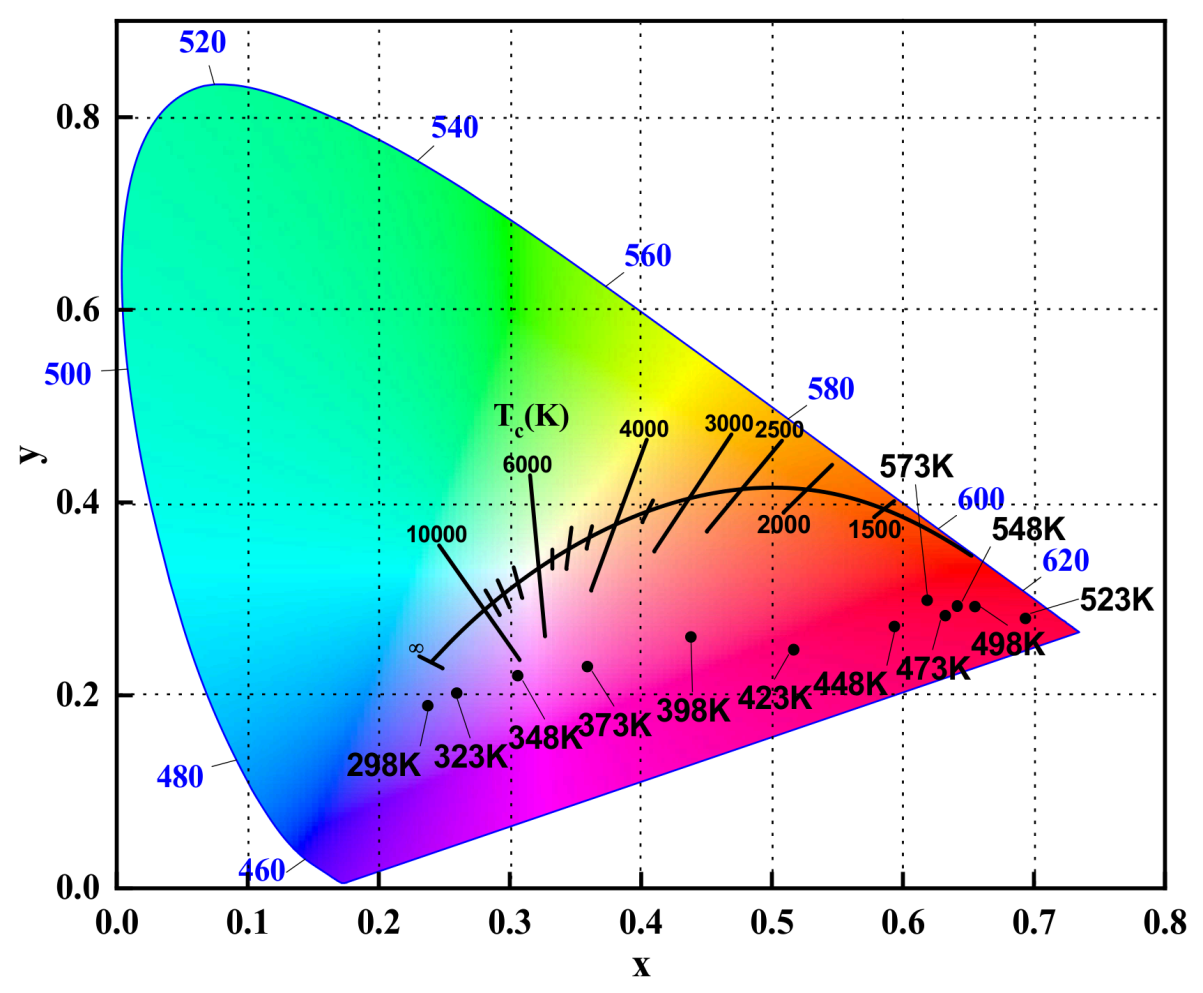
The CIE coordinates of the 1% Tm^3+^, 6% Yb^3+^: Gd_0.2_Bi_1.8_WO_6_ phosphor at different temperatures.

The CIE colour coordinates and colour temperatures of this powder at different temperatures are shown in Figure 15. As the temperature increases (298–573 K), the chromaticity coordinates of the material shift from the blue region to the red region.

**Figure 16:**
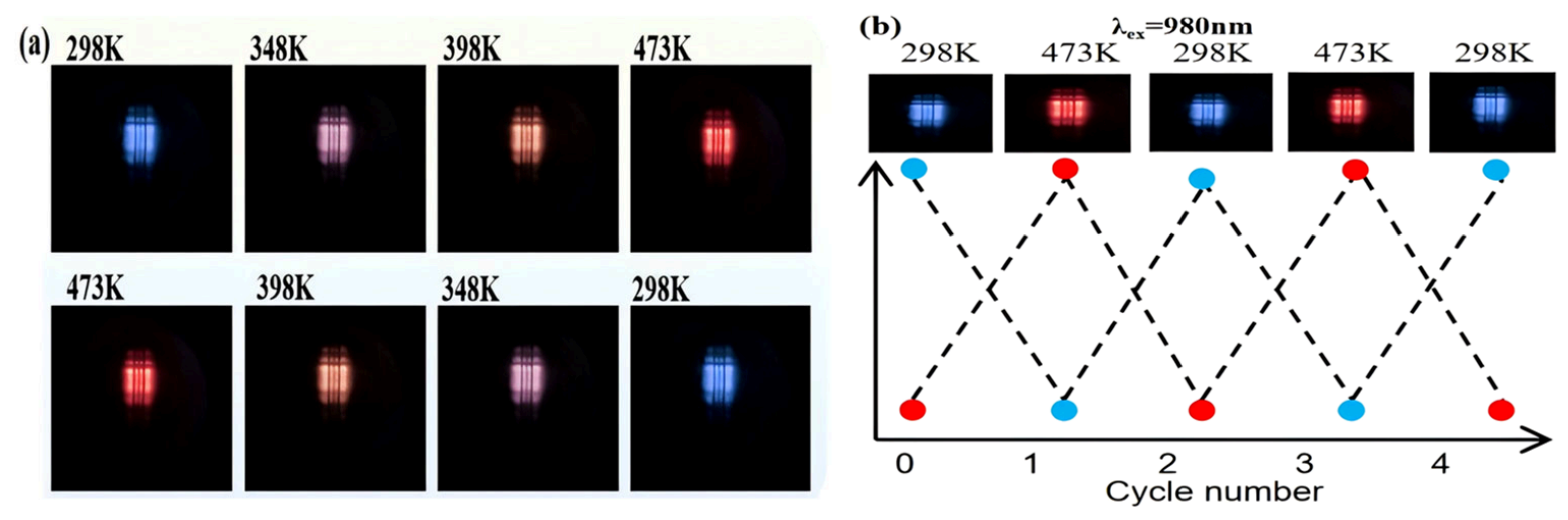
(**a**) Thermochromic photos of 1% Tm^3+^, 6% Yb^3+^: Gd_0.2_Bi_1.8_WO_6_ phosphor under different temperature at 980 nm excitation; (**b**) luminescence color change of under the temperature cycles.

Thermochromic photos of this phosphor in a dark environment are shown in Figure 16. When the sample is irradiated with a 980 nm (518 mW) laser, as the temperature increases (298–473 K), the luminescence colour of the sample changes from blue to red. According to spectrum 11 (a), at room temperature, the blue light intensity emitted by the sample at 476 nm is not significantly different from the red light intensity at 683 nm and 703 nm. When the temperature rises from room temperature to 473 K, the red light emitted by the sample rapidly increases at 683 nm and 703 nm, but the blue light rapidly weakens, causing the composite light color to shift towards red light. When the temperature exceeds 473 K, the red light also begins to weaken, so the corresponding color of the light begins to shift in the opposite direction. When the temperature gradually decreases from 573 K, the luminescence colour of the sample changes from red to blue, which indicates that the phosphor synthesized in this work has potential application value in thermochromism and laser anticounterfeiting. After four cycles of heating and cooling, the luminescence of the sample switched between blue and red light, and the luminescence was relatively stable.

### 2.7. Magnetic Properties of the Materials

Considering that Gd^3+^ has seven unpaired 4f electrons and strong paramagnetic properties, it can significantly shorten the relaxation time of surrounding water molecules at magnetic resonance T1 and generate T1-weighted signals, and its chelates can serve as good magnetic resonance contrast agents. If both fluorescence temperature measurements and magnetic resonance imaging can be achieved with synthetic materials, their application value will increase. Therefore, the magnetic properties of synthetic materials at different temperatures were analysed.

**Figure 17:**
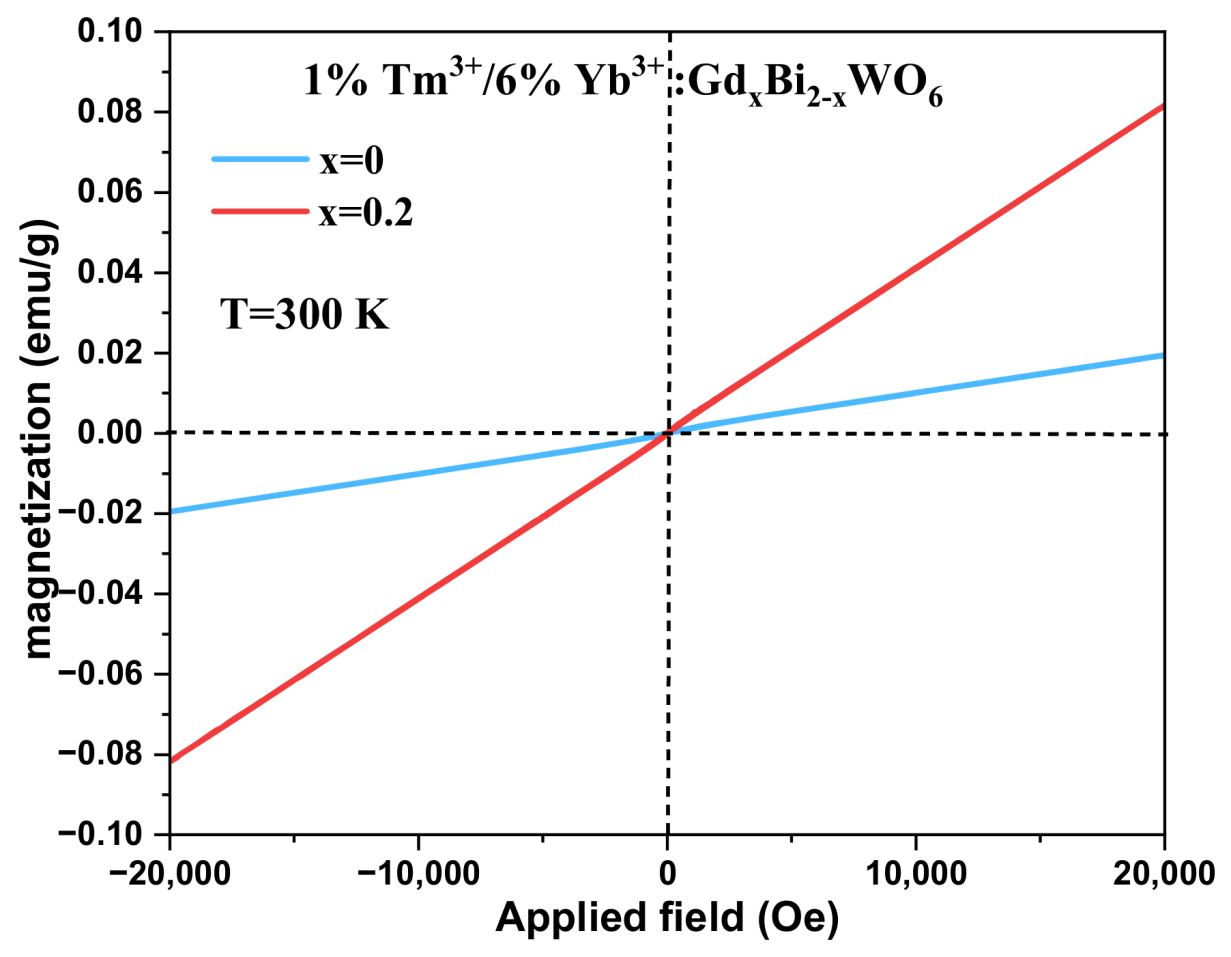
Magnetization intensity of the 1% Tm^3+^, 6% Yb^3+^: Gd_x_Bi_2−x_WO_6_ (x = 0, 0.2) powder under different external magnetic fields at 300 K.

As shown in Figure 17, when the applied magnetic field increased from −20 to 20 kOe, the magnetization strength of 1% Tm^3+^, 6% Yb^3+^, x% Gd: Bi_2_WO_6_ (x = 0, 10) powder under an external magnetic field significantly increased. Compared with that of the Tm^3+^, Yb^3+^: Bi_2_WO_6_ powder, the magnetization of the sample doped with Gd^3+^ (x = 0.2) increased fourfold and reached 0.08174 emu/g (20 kOe, 300 K).

Table 8 presents a comparison of the magnetic properties of different matrix materials doped with Gd^3+^. The 1% Tm^3+^, 6% Yb^3+^: Gd_0.2_Bi_1.8_WO_6_ powder particles synthesized in this work exhibit typical paramagnetic properties.

**Figure 18:**
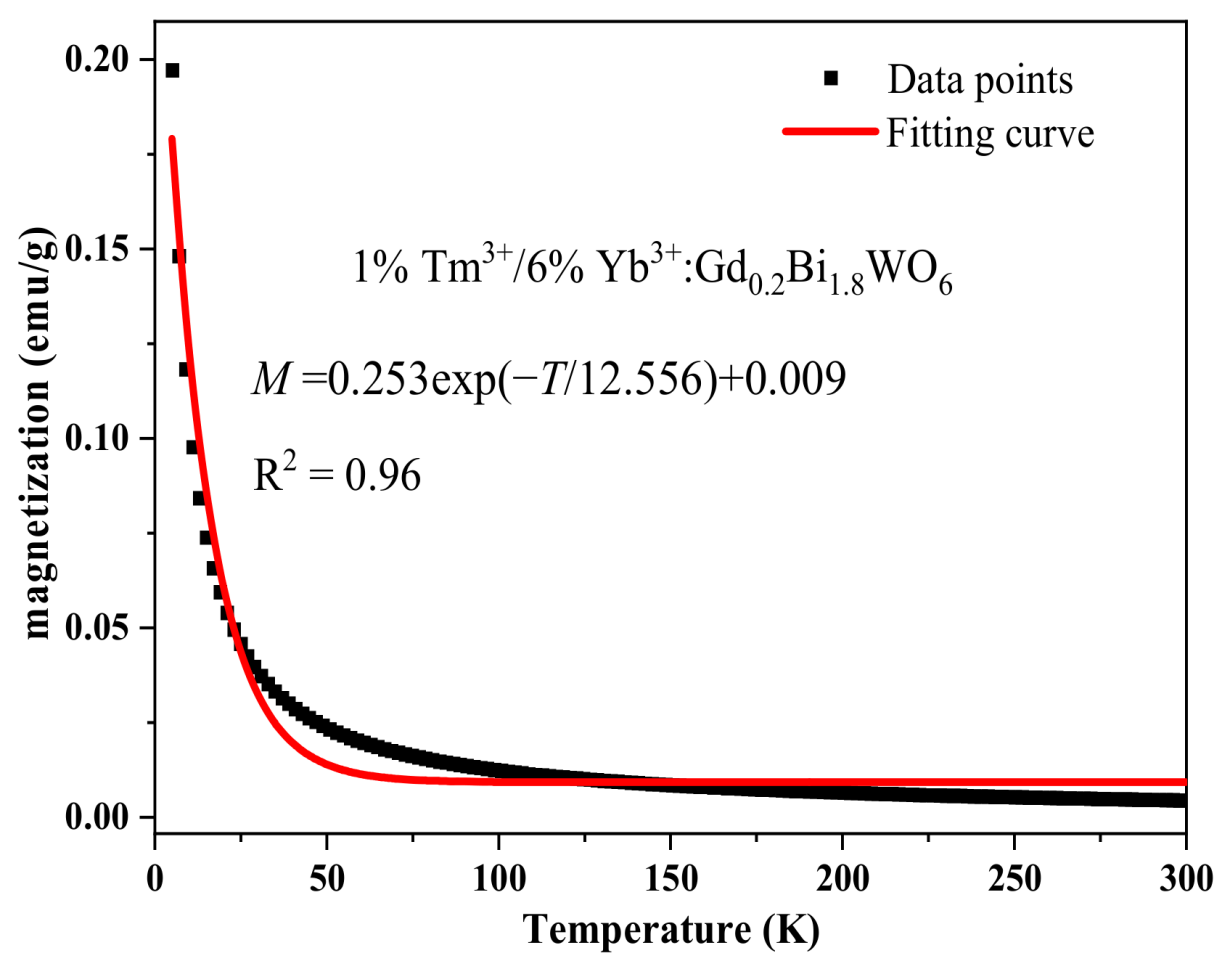
Magnetism (5~300 K) of the 1% Tm^3+^, 6% Yb^3+^: Gd_0.2_Bi_1.8_WO_6_ powder samples under an external magnetic field of 1 kOe.

The temperature-dependent magnetic properties of the 1% Tm^3+^, 6% Yb^3+^: Gd_0.2_Bi_1.8_WO_6_ powder under an external magnetic field of 1 kOe are shown in Figure 18. At 1 kOe, as the temperature increases from 5 K to 300 K, the magnetization intensity of this powder samples decreases exponentially. The data were fit to obtain the relationship between the magnetization *M* and temperature *T* of the powder sample as follows:

(27) M =0.253exp(−T/12.556)+0.009

In Figure 18, R^2^ = 0.96 represents the degree of agreement between the theoretical fit and the experimental value. Through the above *M*–*T* function relationship, this material can provide information regarding magnetization intensity under external magnetic fields while enabling temperature measurements, thereby permitting the dual calibration of temperature and magnetism. Under an external magnetic field of 1 kOe and temperatures of 5 K and 300 K, the magnetization strengths of the 1% Tm^3+^, 6% Yb^3+^: Gd_0.2_Bi_1.8_WO_6_ samples were 0.19724 and 0.00447 emu/g, respectively. The above results indicate that the powder synthesized in this work has potential applications in fluorescence thermometry and magnetic resonance imaging.

## 3. Materials and Methods

### 3.1. Theoretical Calculation

All density functional theory (DFT) calculations were conducted with the Vienna ab-Initio Simulation Package (VASP) [40,41]. The generalized gradient approximation (GGA) using the Perdew–Burke–Ernzerhof (PBE) functional was employed to account for exchange correlations. The ionic cores and valence electrons were described using the projected augmented wave (PAW) method. A plane-wave basis set with an energy cutoff of 450 eV was adopted for the calculations. On the basis of a 2 × 1 × 2 supercell with 144 atoms, Bi_2_WO_6_ models with different concentrations of doped Tm, Yb, and Gd were constructed. Additionally, 2 × 2 × 2 gamma-centre grids were used for structure relaxation. During structure optimization, the conjugate gradient method was used, and the convergence criteria for energy and force were set to 1 × 10^−5^ eV and 2 × 10^−2^ eV Å^−1^, respectively.

### 3.2. Experimental Methods

The raw materials Bi_2_O_3_ (99.9%), WO_3_ (99.9%), Tm_2_O_3_ (99.9%), Yb_2_O_3_ (99.9%), and Gd_2_O_3_ (99.9%) (from Aladdin, Shanghai, China) were weighed using an AL104 electronic balance and mixed in accordance with the chemical ratios of y% Tm^3+^, 6% Yb^3+^: Gd_x_Bi_2−x_WO_6_ (x = 0, 0.06, 0.1, 0.2, 0.3; y = 0.5, 1, 3, 6). The ion doping concentration is expressed as a mole percentage concentration. The powder was then ground for 25 min and calcined in a muffle furnace (SX-12-16) at 800 °C for 3 h in air to obtain various Tm^3+^, Yb^3+^, Gd^3+^: Bi_2_WO_6_ samples, and the above doping concentrations were determined on the basis of the amounts of the materials.

Physical phase analysis of the samples was performed using a X-ray diffractometer (XRD-6100, Shimadzu, Kyoto, Japan). Scanning electron microscope (ZEISS SUPRA55 VP, Carl Zeiss, Jena, Germany) was used to observe the microscopic morphology, size and dispersion of the samples. The elemental and powder contents on the surface were analysed using a EDS X-ray energy spectrometer (X-Flash SDD 5010, Bruker, Karlsruhe, Germany). Fluorescence detection was performed using an full-featured steady-state/transient fluorescence spectrometer (Edinburgh VFLS920, Edinburgh Instruments, Livingston, UK), which was used to measure the upconversion emission spectra of the samples at different pump powers, and the excitation source was infrared light from a 980 nm laser (MDL-III-980-2W, Edinburgh Instruments, UK). Diffuse reflectance spectra were obtained with a UV–visible diffuse reflection spectrometer (UV-3900 plus, Shimadzu, Japan). To detect the upconversion luminescence of the samples at different temperatures, the samples were heated in the high-temperature accessory heater of the spectrometer to temperatures ranging from 298 K to 573 K. The magnetic properties of the synthesized powders were measured using an high precision magnetic measurement system (MPMS XL-7 SQUID, San Diego, CA, USA). The magnetic changes in the synthesized powder were measured at a temperature of 300 K under an external magnetic field ranging from −20 to 20 kOe. The temperature-dependent magnetic properties of the synthesized powders were measured in the temperature range of 5–300 K under an external magnetic field of 1 kOe. To avoid laser-induced thermal effects, the samples were excited with a low excitation power. The instruments were calibrated before use.

## 4. Conclusions

y% Tm^3+^, 6% Yb^3+^: Gd_x_Bi_2−x_WO_6_ (x = 0, 0.06, 0.1, 0.2, 0.3; y = 0.5, 1, 3, 6) upconversion luminescent materials were prepared via a high-temperature solid-phase method and calcined at 800 °C for 3 h. Young’s modulus and the Debye temperature of the Bi_2_WO_6_ matrix material doped with Gd^3+^ were calculated to be 183.579 GPa and 392.45 K, respectively. Compared with the NaYF_4_ matrix, the Bi_2_WO_6_ material doped with Gd^3+^ had lower Debye temperatures, which resulted in a lower probability of nonradiative transitions. The XRD results revealed that the doping of low-concentration small-radius ions Tm^3+^ and Yb^3+^ does not affect the orthorhombic crystal structure of the Bi_2_WO_6_ matrix, and its diffraction peaks shift towards larger angles with increasing doping concentrations. However, as the amount of doped Gd^3+^ increases, diffraction peaks of Gd_2_WO_6_ appear, and the main diffraction peak of the lattice (131) shifts to a smaller angle, indicating that in addition to Gd^3+^ replacing Bi^3+^, excessive Gd^3+^ ions enter the main lattice gap, thereby expanding the lattice. SEM images revealed that the samples were composed of 0.5~3 μm heterogeneous particles, and EDS analysis revealed that the elements were evenly distributed in the powder. Under 980 nm excitation, the emission intensity of the 1% Tm^3+^, 6% Yb^3+^: Gd_0.2_Bi_1.8_WO_6_ sample was the strongest among all the samples, and the intensity of the Tm^3+^ peak at 804 nm increased by 81.9 times. Moderate Tm^3+^, Yb^3+^ and Gd^3+^ doping can cause the absorption band of the synthesized material to shift towards longer wavelengths, and the band gaps decrease. When the sample was excited at 980 nm (178–518 mW), the logarithm of its light intensity and power exhibited a linear relationship. Tm^3+^ was emitted from three-photon absorption at 476 nm (n = 2.51) and from two-photon absorption at 683, 703, 779, and 804 nm (n = 1.89 1.97, 1.73 and 1.67, respectively). Under 980 nm excitation (518 mW), from 298 K to 573 K, on the basis of the fluorescence intensity ratio of 703 nm/804 nm or 703 nm/476 nm for the 1% Tm^3+^, 6% Yb^3+^: Gd_0.2_Bi_1.8_WO_6_ sample, the maximum relative sensitivities reach 0.0153 K^−1^ at 298 K and 3.4907 K^−1^ at 298 K, with minimum temperature resolutions of 0.0474 K at 423 K and 0.0083 K at 298 K. Under excitation at 980 nm, as the temperature increased from 298 K to 473 K, the luminescence colour of this powder changed from blue to purple, then to orange, and finally to red. Under an external magnetic field of 20 kOe and a temperature of 300 K, the magnetization of the sample was 0.08174 emu/g. Compared with that of the undoped Gd^3+^ sample, the temperature sensitivity of the Gd^3+^-codoped sample increased by 92.35 times. This material enables noncontact, high-sensitivity micro/nanoscale temperature sensing and real-time magnetic feedback, with potential applications in temperature sensing and magnetic resonance imaging.

## Figures and Tables

**Table 1:** Lattice parameters and defect formation energy of samples calculated by VASP.

Host	Ion Doping Mode	a/Å	b/Å	c/Å	V/Å	Defect Formation Energy (eV)
Bi_2_WO_6_	-	5.517	16.671	5.519	507.685	-
	10% Tm^3+^ and 6% Yb^3+^ substitution for Bi^3+^ sites	5.504	16.565	5.516	502.908	-
	10% Tm^3+^, 6% Yb^3+^ and 10% Gd^3+^ substitution for Bi^3+^ sites	5.510	16.477	5.516	500.848	−44.124
	10% Tm^3+^, 6% Yb^3+^ substitution for Bi^3+^ sites, and 10% Gd^3+^ interstitial doping	5.519	16.750	5.534	511.715	−24.526

**Table 2:** Calculation results of the elastic coefficients C_ij_.

Matrix	C_ij_	C_11_	C_22_	C_33_	C_44_	C_55_	C_66_	C_12_	C_13_	C_23_
Bi_2_WO_6_	Value	209.92	184.01	198.32	76.91	75.07	95.06	76.09	33.53	53.60
Gd_0.2_Bi_1.8_WO_6_	Value	213.09	185.15	198.03	74.03	75.02	95.07	78.04	36.83	53.20

**Table 3:** Calculation parameters and Young’s modulus *E* of the Bi_2_WO_6_ host and Gd_0.2_Bi_1.8_WO_6_.

Calculation Parameters	Bi_2_WO_6_	Gd_0.2_Bi_1.8_WO_6_
Bulk Modulus: B	101.858 GPa	103.324 GPa
Shear Modulus: G	76.845 GPa	76.245 GPa
Longitudinal wave velocity: *V_p_*	4634.440 m/s	4663.646 m/s
Transverse wave velocity: *V*_s_	2842.177 m/s	2844.275 m/s
Young’s modulus: *E*	184.210 GPa	183.579 GPa

**Table 4:** Debye temperatures and phonon energies of typical host materials.

Material	Debye Temperature *θ*_D_	Acquisition Method	Ref	Phonon Energy	Ref
NaYF_4_	532 K	DFT	[20]	≈350 cm^−1^	[21]
Bi_2_WO_6_	391.94 K	DFT	This Work	≈300 cm^−1^	[22]
Gd_0.2_Bi_1.8_WO_6_	392.45 K	DFT		/	/

**Table 5:** Cell parameters of samples obtained by JADE.

Sample	hkl	131	060	202	133	262	204	a (Å)	b (Å)	c (Å)	V (Å^3^)
Bi_2_WO_6_	*D*_hkl_	820	330	389	270	882	222	5.472	16.389	5.461	489.870
	FWHM	0.152	0.274	0.239	0.367	0.250	0.488				
1% Tm^3+^, 6% Yb^3+^: Bi_2_WO_6_	*D*_hkl_	759	318	394	264	879	215	5.470	16.393	5.451	488.850
	FWHM	0.150	0.279	0.242	0.355	0.142	0.486				
1% Tm^3+^, 6% Yb^3+^: Gd_0.1_Bi_1.9_WO_6_	*D*_hkl_	836	330	401	281	806	255	5.468	16.394	5.440	488.920
	FWHM	0.141	0.270	0.238	0.355	0.151	0.414				
1% Tm^3+^, 6% Yb^3+^: Gd_0.2_Bi_1.8_WO_6_	*D*_hkl_	852	332	399	272	913	256	5.4446	16.420	5.465	488.960
	FWHM	0.139	0.276	0.239	0.345	0.141	0.412				
1% Tm^3^, 6% Yb^3+^: Gd_0.3_Bi_1.7_WO_6_	*D*_hkl_	769	315	392	261	867	252	5.453	16.408	5.460	488.520
	FWHM	0.146	0.281	0.242	0.358	0.145	0.419				

**Table 6:** Energy level lifetimes of the 1% Tm^3+^, 6% Yb^3+^: Gd_x_Bi_2−x_WO_6_ (x = 0, 0.2) samples.

Sample	λex	Energy Level	λem	τ/μs
1% Tm^3+^, 6% Yb^3+^: Bi_2_WO_6_	980 nm	^3^F_3_→^3^H_6_	703 nm	12.53
		^3^H_4(2)_→^3^H_6_	804 nm	19.41
1% Tm^3+^, 6% Yb^3+^: Gd_0.2_Bi_1.8_WO_6_		^3^F_3_→^3^H_6_	703 nm	12.80
		^3^H_4(2)_→^3^H_6_	804 nm	25.34

**Table 7:** Temperature measurement sensitivity of rare-earth ions in different matrix materials.

Material	Energy Level	Temperature/K	Maximum *S_a_*/K^−1^	Maximum *S_r_*/K^−1^	Ref.
Tm^3+^, Yb^3+^, Gd^3+^: Y_2_O_3_	(Tm^3+^)^1^G_4(a)_/^1^G_4(b)_→^3^H_6_	298–533	0.0013@298 K	/	[33]
Tm^3+^, Yb^3+^, Gd^3+^, Er^3+^: CaWO_4_	(Tm^3+^)^3^H_4_→^3^H_6_/ (Er^3+^)^2^H_11/2_→^4^I_15/2_	303–513	0.0936@303 K	0.015@303 K	[7]
Yb^3+^, Ho^3+^, Gd^3+^: CaF_2_	(Ho^3+^)^5^F_4_+^5^S_2_/^5^F_5_→^5^I_8_	323–498	0.00178@323 K	0.0107@498 K	[8]
Ho^3+^, Gd^3+^, Yb^3+^: NaYF_4_	(Ho^3+^)^5^F_5_/^5^F_4_+^5^S_2_→^5^I_8_	298–548	0.00009@548 K	0.0127@298 K	[34]
Gd^3+^, Yb^3+^, Er^3+^, Tm^3+^: NaYF_4_	(Tm^3+^)^3^H_4_→^3^H_6_/ (Er^3+^)^2^H_11/2_→^4^I_15/2_	293–523	0.039@527 K	0.0107@409 K	[35]
Tm^3+^, Yb^3+^: Bi_2_WO_6_	(Tm^3+^) ^3^F_2,3_→^3^H_6_ ^3^F_3_→^3^H_6_/^1^G_4_→^3^F_4_	298–573 298–573	0.00254@298 K 0.167@573 K	0.00144@298 K 0.0378@298 K	[15]
Tm^3+^, Yb^3+^: Gd_0.2_Bi_1.8_WO_6_	(Tm^3+^) ^3^F_3_→^3^H_6_/^3^H_4_→^3^H_6_	298–573	0.000084@573 K	0.0153@298 K	This work
	(Tm^3+^) ^3^F_3_→^3^H_6_/^1^G_4_→^3^H_6_	298–573	61.3556@573 K	3.4907@298 K	

**Table 8:** Magnetization of the different host materials.

Material	Applied Field/kOe	Maximum Magnetization/emu/g	Ref.
20% Tb^3+^, 11% Gd^3+^: CeF_3_	−20~20	0.0623 (300 K)	[37]
Eu^3+^/7% Gd^3+^: ZrO_2_	−2~2	0.068 (300 K)	[38]
3% Yb^3+^, 0.2% Ho^3+^,10% Ag^+^: GdF_3_	−10~10	1.3 (300 K)	[39]
20% Yb^3+^, 1% Ho^3+^, 15% Gd^3+^: CaF_2_	−20~20	0.95 (300 K)	[8]
1% Tm^3+^, 6% Yb^3+^: Bi_2_WO_6_	−20~20	0.01951 (300 K)	This work
1% Tm^3+^, 6% Yb^3+^: Gd_0.2_Bi_1.8_WO_6_	−20~20	0.08174 (300 K)	
	1	0.19724 (5 K)	
	1	0.00447 (300 K)	

## Data Availability

The data that supports the findings of this study are available on request from the corresponding author.
